# Anteroposterior axis patterning by early canonical Wnt signaling during hemichordate development

**DOI:** 10.1371/journal.pbio.2003698

**Published:** 2018-01-16

**Authors:** Sébastien Darras, Jens H. Fritzenwanker, Kevin R. Uhlinger, Ellyn Farrelly, Ariel M. Pani, Imogen A. Hurley, Rachael P. Norris, Michelle Osovitz, Mark Terasaki, Mike Wu, Jochanan Aronowicz, Marc Kirschner, John C. Gerhart, Christopher J. Lowe

**Affiliations:** 1 Institut de Biologie du Développement de Marseille, Aix-Marseille Université, CNRS UMR 7288, Marseille, France; 2 Hopkins Marine Station, Department of Biology, Stanford University, Pacific Grove, California; 3 Department of Organismal Biology and Anatomy, University of Chicago, Chicago, Illinois; 4 Department of Cell Biology, University of Connecticut Health Center, Farmington, Connecticut; 5 Department of Natural Sciences, St. Petersburg College, Clearwater, Florida; 6 Department of Molecular and Cellular Biology, University of California Berkeley, Berkeley, California; 7 Department of Systems Biology, Harvard Medical School, Boston, Massachusetts; University of Florida, United States of America

## Abstract

The Wnt family of secreted proteins has been proposed to play a conserved role in early specification of the bilaterian anteroposterior (A/P) axis. This hypothesis is based predominantly on data from vertebrate embryogenesis as well as planarian regeneration and homeostasis, indicating that canonical Wnt (cWnt) signaling endows cells with positional information along the A/P axis. Outside of these phyla, there is strong support for a conserved role of cWnt signaling in the repression of anterior fates, but little comparative support for a conserved role in promotion of posterior fates. We further test the hypothesis by investigating the role of cWnt signaling during early patterning along the A/P axis of the hemichordate *Saccoglossus kowalevskii*. We have cloned and investigated the expression of the complete Wnt ligand and Frizzled receptor complement of *S*. *kowalevskii* during early development along with many secreted Wnt modifiers. Eleven of the 13 Wnt ligands are ectodermally expressed in overlapping domains, predominantly in the posterior, and Wnt antagonists are localized predominantly to the anterior ectoderm in a pattern reminiscent of their distribution in vertebrate embryos. Overexpression and knockdown experiments, in combination with embryological manipulations, establish the importance of cWnt signaling for repression of anterior fates and activation of mid-axial ectodermal fates during the early development of *S*. *kowalevskii*. However, surprisingly, terminal posterior fates, defined by posterior *Hox* genes, are unresponsive to manipulation of cWnt levels during the early establishment of the A/P axis at late blastula and early gastrula. We establish experimental support for a conserved role of Wnt signaling in the early specification of the A/P axis during deuterostome body plan diversification, and further build support for an ancestral role of this pathway in early evolution of the bilaterian A/P axis. We find strong support for a role of cWnt in suppression of anterior fates and promotion of mid-axial fates, but we find no evidence that cWnt signaling plays a role in the early specification of the most posterior axial fates in *S*. *kowalevskii*. This posterior autonomy may be a conserved feature of early deuterostome axis specification.

## Author summary

The anteroposterior (A/P) axis is a conserved feature of bilateral animals and is defined in the anterior by a head and in the posterior by a trunk. The secreted family of Wnt proteins and their antagonists play a conserved role in setting up this axis during early development in many bilaterians. The widely accepted model for the role of Wnt signalling in A/P axis specification is by the establishment of a simple activity gradient whereby high levels of Wnt lead to posterior fates and low levels to anterior fates. In this study we further test this model, examining the role of Wnt signaling in the acorn worm, a representative of hemichordates that belongs to the superphylum Deuterostomia along with chordates. We find strong evidence supporting the hypotheses that Wnt signaling represses anterior fates and promotes more caudal fates in the ectoderm, in a similar way to central nervous system development in vertebrates However, we find that the most posterior territory is established independently of Wnt signalling, which is inconsistent with the prevailing model of Wnt function in A/P patterning. We conclude that these data are not inconsistent with vertebrate patterning data and may represent a conserved feature of deuterostome axis patterning.

## Introduction

The Wnt family of secreted ligand proteins is involved in a wide range of developmental functions during animal development, from embryonic induction to cell fate specification and the generation of cell polarity [1,2]. The presence of Wnt ligands, receptors, and Wnt antagonists in cnidarians, sponges, and ctenophores indicates an early origin and diversification of the Wnt pathway during the radiation of metazoan phyla, before the emergence of bilaterians [3–7]. Wnt ligands can act via noncanonical and canonical pathways. The canonical Wnt (cWnt) or Wnt-β-catenin pathway is the best studied and involves ligand binding of both the Frizzled (Fz) receptor and Lrp5/6 coreceptor. Activation of this pathway leads to stabilization of β-catenin and trafficking to the nucleus where it activates downstream targets in cooperation with T-cell factor/lymphoid enhancer factor (Tcf/lef) transcription factors. Comparative studies have proposed conserved roles of cWnt signaling in basic axial patterning of metazoan embryos, suggesting that cWnt signaling played a fundamental role in the early establishment of metazoan axis formation [3,4,8–14].

cWnt plays critical axial patterning roles during the development of metazoan embryos: in cnidarians it is first involved in the establishment of the endoderm with a pulse of β-catenin in the animal pole during early animal/vegetal (AV) patterning, then later to define the polarity of the oral/aboral axis of the adult. In bilaterians, there is robust evidence from both deuterostomes and lophotrochozoans of a similar role of β-catenin in AV patterning [15–17], suggesting this is a widely conserved mechanism in eumetazoans. Perhaps the best known axial patterning role of cWnt is during the establishment of the anteroposterior (A/P) axis, with compelling comparative data generated across bilaterian lineages [11]. However, these comparative data do not represent a single conserved developmental role of cWnts in A/P patterning and can be divided into two discrete phases that are mechanistically distinct; the first in establishment of the A/P axis, and second, after the A/P axis has been established, the initiation of axis elongation from a posterior growth zone [18].

Within the bilaterians, most of the data on the early role of cWnt signalling in the establishment of A/P pattern is from vertebrates. cWnt signaling has its strongest effect on head patterning, acting as a classical morphogen; it both represses anterior (forebrain and midbrain) neural fates and induces posterior (hindbrain) neural fates in a dose-dependent manner [19–24]. Wnt antagonists, such as Dickkopf (Dkk) and Secreted frizzled-related proteins (Sfrps), expressed in the anterior neurectoderm and in the prechordal plate mesoderm of the organizer, protect the anterior neurectoderm from the posteriorizing effects of Wnt ligands secreted from the mesoderm and posterior neural plate [21,25,26]. It has thus been proposed that a simple gradient of Wnt activity—high in the posterior and low in the anterior—endows cells with positional information along the A/P axis in the central nervous system [10,20,23]. Data from several species strongly support this model of brain patterning. However, surprisingly, the role of cWnt signaling in the establishment of the most posterior region of the neural plate has not been extensively investigated, and most focus has been on the effect of Wnts in repression of forebrain and midbrain and promotion of hindbrain fates, with little relevant data on the spinal cord [27]

Experimental results from nonvertebrate chordates, the cephalochordates and tunicates, are consistent with data from vertebrates, but suggest that this patterning system is not as fully deployed as it is in vertebrates. In the cephalochordate, *Branchiostoma floridae*, Wnt ligands are localized posteriorly and antagonists anteriorly [28–30]. However, constitutive activation of the cWnt pathway using glycogen synthase kinase 3 beta (GSK3β) inhibitors only represses far anterior markers and expands only blastoporal markers, suggesting that Wnt signaling determines the identity of the two ends of the embryo, but not the intervening regions that are responsive to cWnt in vertebrates [29]. The role of Wnts in early ascidian development remains largely unexplored experimentally. However, the expression of *wnt5* posteriorly and the anterior localization of negative regulators such as *sfrp1/5* and *ror* are suggestive of a potential role in A/P patterning [31–33]. Outside of chordates, the role of Wnts in A/P patterning has recently been demonstrated in sea urchins during larval development [34,35] and has drawn comparisons with cWnt suppression in the anterior neural plate of vertebrates, suggesting common elements of regulation between the apical pole of sea urchin larvae and the anterior neural plate of chordates [36].

In protostomes, further broad phylogenetic support for an ancient role of Wnt in A/P patterning comes from representatives in ecdysozoans and lophotrochozoans. In lophotrochozoans, this is particularly striking during regeneration in planarians demonstrating a critical role of β-catenin in the decision between regeneration of head or tail following experimental amputations and during homeostasis in maintenance of the posterior [37–39]. In the annelid *Platynereis dumerilli*, cWnt activation during early embryonic development results in repression of anterior markers [40]. In arthropods, there is no early axial role of cWnt signaling in *Drosophila*. However, in the basal short germ band insect, *Tribolium*, analysis of axin, a cWnt signaling repressor, reveals an important role of Wnts in defining the early A/P axis [41]. When considered with the polarized expression of Wnts and their antagonists during *Caenorhabditis elegans* early development, an ancestral role of cWnt in ecdysozoans A/P development is implied [42]. A recent study of regeneration in a representative acoel, a group that most likely occupies a key phylogenetic position before the protostome/deuterostome split [43] reveals a key role of cWnt in regeneration, very similar to planarians.

With the aim of adding an important additional data point to the function of cWnt signaling in A/P axis formation of deuterostome and bilaterians, we have investigated its involvement in specifying embryonic axial properties during the early development of the direct-developing enteropneust *S*. *kowalevskii*. Hemichordates are the sister group to echinoderms and are closely related to chordates [44–46]. They occupy a key phylogenetic position for addressing hypotheses of early deuterostome evolution. Previous studies in *S*. *kowalevskii* have demonstrated close transcriptional and signaling similarities with vertebrates during early A/P patterning of ectodermal development. The enteropneust body plan is divided into three main domains: a prosome/proboscis that is transcriptionally similar to the vertebrate forebrain, a mesosome/collar, similar to a midbrain, and a metasome/trunk, similar to a hindbrain and spinal cord [47,48]. It is this transcriptional network involved in ectodermal regionalization that is regulated by cWnt signaling in vertebrates and raises the obvious question of whether the establishment of the network is similarly regulated by cWnt in enteropneusts. An earlier study demonstrated that β-catenin is a critical component of AV patterning and plays a central role in specifying the endomesoderm [15] in a manner very similar to its early role in echinoderm and ascidian development. We also demonstrated that the early endomesoderm subsequently acts as an early organizer and defines the posterior of the embryo. Preliminary observations from this work revealed that the cWnt pathway was clearly an important component of early A/P patterning. The present manuscript explicitly investigates the role of cWnt signaling in the early specification of the A/P axis. Using a range of experimental approaches, we find strong support for a conserved role of cWnt signaling in the early establishment of the A/P axis in *S*. *kowalevskii*. We find three distinct regions in the ectoderm with differing responses to cWnt; first, as in many animals, cWnt signaling inhibits formation of the anterior region by down-regulation of anterior genes of the proboscis and anterior collar. Second, similar to vertebrates, it promotes mid-axial fates by up-regulating genes of the posterior collar, anterior, and mid-trunk. However, surprisingly the terminal posterior ectodermal domain around the blastopore, defined by overlapping expression domains of many Wnt ligands, is initially insensitive to cWnt signaling. We discuss the comparative implications of these findings. Our experiments give robust support for the hypothesis that this pathway was involved in early A/P axis specification deep in bilaterian evolution, predating the diversification of the deuterostome phyla. It also challenges the paradigm of a simple cWnt gradient involved in the specification of the entire A/P axis, and raises the possibility that the most posterior bilaterian ectodermal territory is specified independent of cWnt.

## Materials and methods

### Animal collection and embryo culturing

Adult *S*. *kowalevskii* were collected intertidally on Cape Cod, MA within the Waquoit Bay Reserve. Animal husbandry and culture techniques were comprehensively described previously [15,49]. Embryos were staged by the normal tables of Bateson [50,51] and Colwin and Colwin [52,53].

### Embryo manipulation and microinjection

Classical embryology experiments and microinjections were carried out as described previously [15]. Targeted blastomere injections were performed under a stereomicroscope using a back-filled needle connected to a glass syringe with plastic tubing. The entire system is filled with mineral oil and the injection is performed manually with the syringe under visual control (injected solution is colored by 1% fast green FCF (F-7252; Sigma-Aldrich, St. Louis, MO). Injection was performed into identified blastomeres at cleavage stages. Injection success was monitored by co-injection of 1% rhodamine dextran (D-1817; Molecular Probes, Eugene, OR). siRNAs targeting β-catenin and Fz5/8 were described previously [15,48]. The open reading frames of *S*. *kowalevskii wnt3*, *sfrp1/5*, and *dkk1/2/4* were cloned into pCS2+, then linearized and used for in vitro synthesis of capped RNA using the SP6 Message Machine kit (Applied Biosystems/Ambion, Foster City, CA). The number of experimental embryos examined is indicated in figure panels.

### Drugs and protein treatments

The cWnt pathway was activated using the GSK-3β inhibitor 1-azakenpaullone [54] (191500; Calbiochem, Sigma-Aldrich) or the recombinant Wnt3a protein (R&D Systems, Minneapolis, MN). Treatments were carried out as described previously [15,55]. The treatments led to robust and homogeneous effects as assessed by in situ hybridization on 2 to 20 embryos per probe.

### Cloning of orthologs

A total of 200,000 expressed sequence tags (ESTs) were screened from six libraries previously described [47,55,56]. Partial sequences were further cloned through library screening using PCR. Genbank accession numbers are as follows: *wnt1*, EU931645; *wnt2*, EU931646; *wnt3*, EU931647; *wnt4*, GU224244; *wnt5*, GU076159; *wnt6*, GU076160; *wnt7*, GU076161; *wnt8*, GU076162; *wnt9*, GU076163; *wnt10*, GU076158; *wnt11*, GU076158; *wnt16*, EU931648.1; *wntA*, GU224245; *fz5/8*, GU075997; *fz1/2/*7, MG711509; *fz4*, MG711510; *fz9/10* MG711511; *dkk1/2/4*, GI:259013424; *sclerostin*, GU076111.1; *sfrp1/5*, GU076117.1; *sfrp3/4* MG682447; *r-spondin*, GU076102.1; *notum*, GU076072.1; *wif*, GU076157.1; *dkk3*, MG682446. Tentative orthology of ESTs was assigned by BLAST. Sequences were aligned with sequences from cnidarians and bilaterians using clustalX [57]. Gene tree analyses were carried out using Bayesean [58] and Neighbor-joining [59] algorithms to assign orthology relationships (See S1 and S2 Figs).

### qPCR

Experimental samples of 40 to 50 embryos each were frozen in liquid nitrogen and stored at −80°C. RNA was extracted using the RNAqueous-Micro Total RNA isolation Kit (Life Technologies, Carlsbad, CA) using a motorized pestle for initial homogenization of samples.

cDNA synthesis was performed with Superscript III (Life Technologies) using 50 ng/μL total RNA in a 20 μL volume, using the oligo(dT)20-primer according to manufacturer’s instructions. qPCR reaction-mix was set up using SsoAdvanced Universal SYBR Green Supermix (Bio-Rad, Hercules, CA) with 0.4 μM final concentration per primer and 0.066 ng/μL final cDNA concentration (cDNA concentration is based on the initial 50 ng/μL total RNA concentration used during cDNA synthesis and dilutions of the cDNA synthesis mix thereafter). qPCR was performed in 96-well plates (Bio-Rad) with 9 μL total reaction volume per well using the CFX-Connect Real-Time System (Bio-Rad) running the following program: 95°C for 3 min for an initial melting, and 95°C for 10 s, 55°C for 40 s for 40 cycles, followed by a melting point analysis.

All primer sets were initially optimized for efficiency at 55°C annealing and low probability of primer-dimer product in No-template controls. All cDNAs were tested in a dilution series to determine the area of linear amplification with a larger set of control primers. cDNAs generally behaved linearly with low standard errors to a final dilution of 0.00833 ng/μL or lower.

Three technical replicates of each sample, a No-Template control, and RT^−^ controls with a subset of primers, were performed for each plate and cDNA. *actin*, *beta-tubulin*, *odc*, and *G3PDH* were used for sample normalization. Actin was used for inter-plate normalization. Quantitative values of gene up- or down-regulation relative to control were calculated using the 2^−ΔΔ CT^ method [60] using the Bio-Rad CFX Manager software version 3.1.

### In situ hybridization

In situ hybridization was carried out as described [47,49]. Stained embryos were post-fixed in 10% formaldehyde in 1 X PBS overnight and then sequentially dehydrated into 100% EtOH, followed by several washes in 100% MeOH before clearing in MurrayClear reagent (2 parts Benzoyl benzoate and 1 part benzyl alcohol) and mounted in Permount. Pictures were taken on a Zeiss Axioimager Z1 using a Zeiss Mrc5 camera. Image panels and figures were constructed with Adobe Photoshop and Adobe Illustrator.

## Results

### Cloning, orthology assignment, and expression of Wnt pathway components

To begin our investigation of the function of cWnt signaling during the development of hemichordates, we cloned and described the expression of key components of the signaling pathway (ligands, receptors, and modifiers) detected by whole mount in situ hybridization and reverse transcription PCR (RT-PCR), over a range of developmental stages, from oocytes to juveniles.

### Wnt ligands

The Wnt ligands are a large family of secreted glycoproteins characterized by an invariant pattern of 22 to 24 highly conserved cysteine residues [61]. We cloned 13 Wnt genes from ESTs sequenced from three developmental stages, from both normalized and non-normalized cDNA libraries [56], which include *wnt1*, *wnt2*, *wnt3*, *wnt4*, *wnt5*, *wnt6*, *wnt7*, *wnt8*, *wnt9*, *wnt10*, *wnt11*, *wnt16*, and *wntA*. All members of the proposed ancestral complement of 13 Wnt subfamilies are present in the hemichordate genome, including Wnt11 and Wnt2 that are absent from the sea urchin genome, and WntA, which has been lost from chordates [62]. We cloned one representative from each Wnt subgroup, and have not detected any paralogy duplicates by systematic screening of ESTs and genome assembly (S1A Fig).

**Fig 1:**
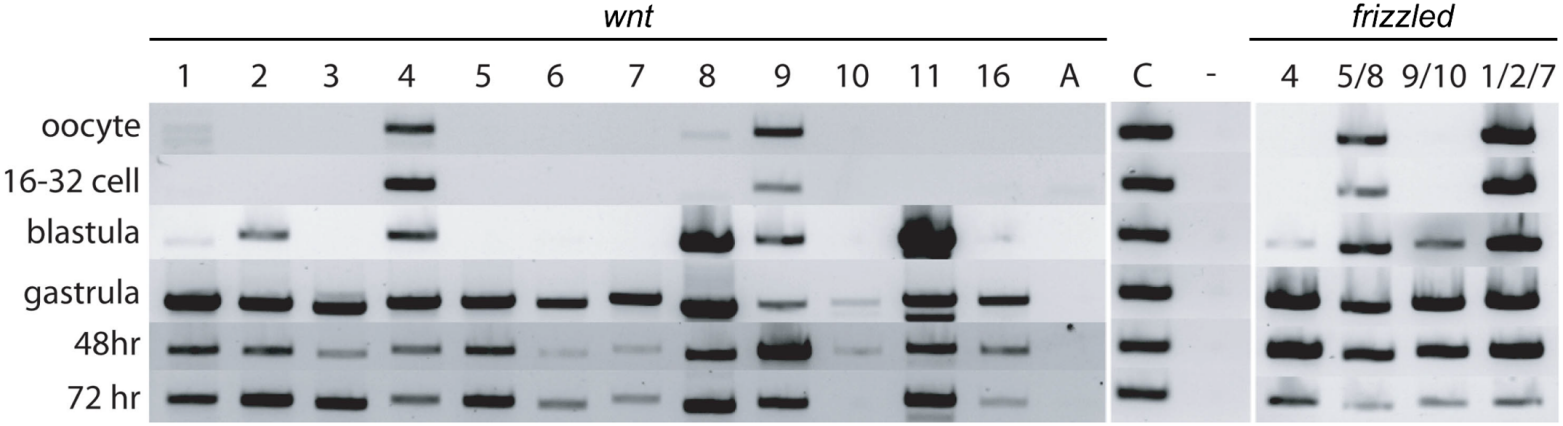
RT-PCR analysis of *Wnt* and *Fz* genes expression during early development. Embryos were harvested at six different stages: oocytes, 16- to 32-cell cleavage stage embryos, late blastula, mid-gastrula, at 48 hpf, and 72 hpf. The first panel shows levels of all 13 *Wnt* genes; the second panel shows the positive control *actin* and a negative control. The third panel shows the levels of the four *Fz* receptor genes. The transcript amounts are comparable across all three panels. *Fz*, frizzled; hpf, h postfertilization; RT-PCR, reverse transcription PCR.

We first present expression data for Wnt ligands by RT-PCR to determine the temporal expression profiles of each gene from oocyte to day 3 of development, when the main features of the hemichordate body plan are established (Fig 1). Maternal expression of two ligands, *wnt4* and *wnt9*, was detected in oocytes and early cleavage stages, and very low levels of *wnt1* and *wnt8* in oocytes. By late blastula/early gastrula and subsequent developmental stages, all Wnts except *wntA* were detectable by RT-PCR.

**Fig 2:**
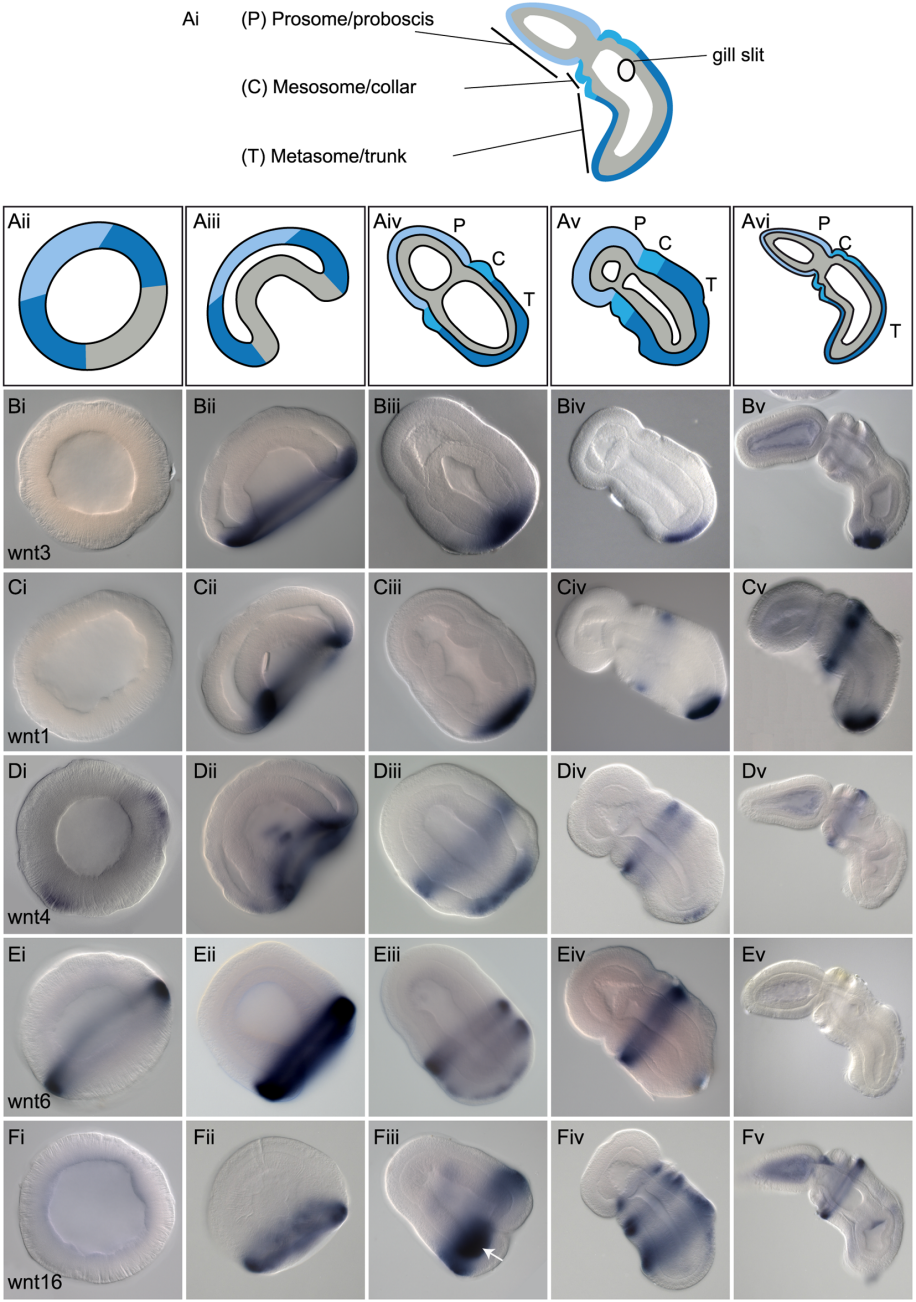
Expression of Wnt genes with blastoporal localization. Whole mount in situ hybridization of Wnt genes with early expression domains around the blastopore. All data are presented as optical sections following clearing in Murray Clear. Developmental staging is from blastula to 72 h of development. All embryos are oriented with anterior or animal (in the case of blastula) to the top left of the panel and posterior or vegetal to the bottom right of the panel. Right column, ventral is to the bottom left. Unless otherwise noted, expression is ectodermal. (A), Schematic representation of optical section through embryos showing the main regions of the embryo representing the major divisions and landmarks of the body plan at 72 hpf (Ai), blastula stages (Aii), gastrula stages (Aiii), 48 h (Aiv), 60 h (Av), and 72 h (Avi) of development with endomesoderm (gray), posterior ectoderm (dark blue), midfate ectoderm (medium blue), and anterior ectoderm (light blue) precursors. (B), Expression of *wnt3*. No expression at blastula (Bi), gastrula side view (Bii), at 48 hpf frontal view (Biii), at 60 hpf in side view (Biv), and at 72 hpf in side view (Bv). (C), Expression of *wnt1* at blastula (Ci), at gastrula stage (Cii), at 48 h frontal section (Ciii), at 60 h side view (Civ), and at 72 h side view (Cv). (D), Expression of *wnt4*, (Di) at blastula, (Dii) at gastrula, (Diii) at 36 h side view, (Div) at 60 h side view, and (Dv) at 72 h side view. (E), Expression of *wnt6*, (Ei) at blastula, (Eii) at gastrula, (Eiii) at 48 h side view, (Eiv) at 60 h side view, and (Ev) at 72 h side view. (F), Expression of *wnt16*, (Fi) at blastula, (Fii) at gastrula, (Fiii) at 48 h side view. The white arrow indicates expression in the ventral posterior endoderm. (Fiv) Expression at 60 h side view, and (Fv) at 72 h side view. hpf, hours postfertilization.

**Fig 3:**
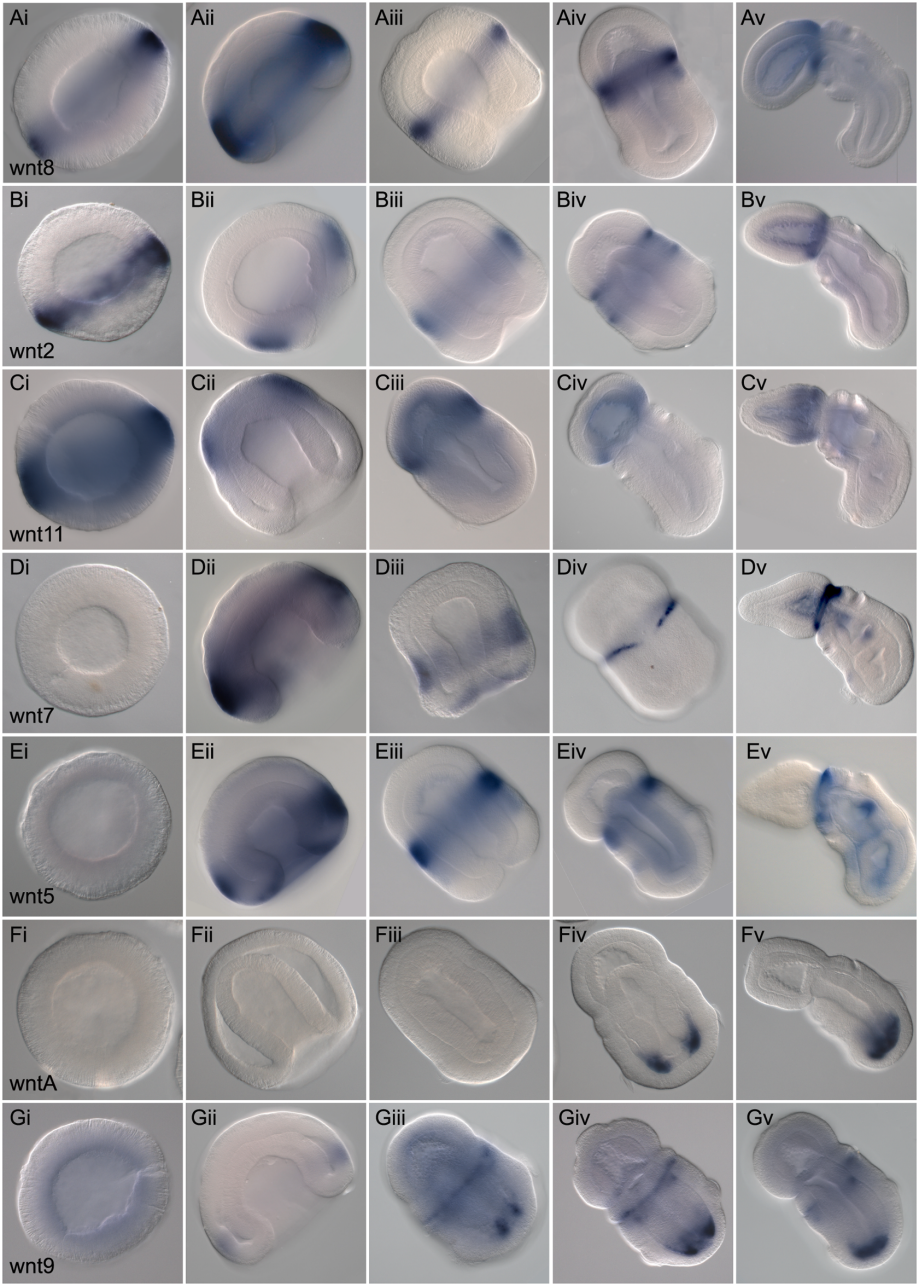
Expression of Wnt genes with anterior ectodermal localizations. Whole mount in situ hybridization of Wnt genes with ectodermal localization in the more anterior domain of the embryo. All data are presented as optical sagittal or frontal sections following clearing in Murray Clear. Developmental staging is from blastula to 72 h of development. All embryos are oriented with anterior, or animal (in the case of blastula), to the top left of the panel and posterior, or vegetal, to the bottom right of the panel. Right column, ventral is to the bottom left. Unless otherwise noted, expression is ectodermal. (Ai-Av), Expression of *wnt8* at blastula stage (Ai), midgastrula stage (Aii), 30 h (Aiii), 50 h, side view (Aiv), and 72 h of development, side view (Av). (Bi-Bv), Expression of *wnt 2*. Blastula stage (Bi), at late gastrula (Bii), at 36 h (Biii), at 48 h, frontal view (Biv), and at 72 h of development, side view (Bv). (Ci-Cv), Expression of *wnt11* at blastula stage (Ci), at gastrula stage (Cii), at 48 h, side view (Ciii), 60 h, side view (Civ), and 72 h of development, side view. (Di-Dv), Expression of *wnt7* at blastula stage (Di), gastrula stage (Dii), 36 h (Diii), 48 h, showing a dorsal view with focal plane through the dorsal ectoderm (Div), and 72 h of development, side view (Dv). (Ei-Ev), Expression of *wnt5* at blastula stage (Ei), at midgastrula stage (Eii), at 48 h, dorsal view (Eiii), at 60 h, side view (Eiv), and at 72 h of development in side view (Ev). (Fi-Fv), Expression of *wntA*, no expression for the first 48 h at blastula stage (Fi), gastrula stage (Fii), and at 40 h of development (Fiii). Expression begins at 48 h shown as a frontal section from a dorsal view (Fiv), and at 72 h of development with a side view (Fv). (Gi-Gv), Expression of *wnt9* at blastula stage (Gi), at midgastrula stage (Gii), at 48 h, showing a ventral view with optical section through the ventral ectoderm and posterior mesoderm (Giii), at 60 h of development again in ventral view (Giv), and at the same stage in side view (Gv).

We describe the patterns of expression for all 13 Wnt genes (with the exception of *wnt10*, which we failed to detect) by whole mount in situ hybridization from mid-blastula (12 hpf [h postfertilization] at 20°C) to day three of development (Figs 2 and 3). The first evidence of zygotic Wnt expression is at mid-blastula: five of the Wnts are detected by in situ hybridization (w*nt4*, *wnt6*, *wnt8*, *wnt2*, *and wnt11*). Their expression is detected in circumferential bands, strongest at the intersection of the AV hemispheres in the region fated to become the blastopore (Figs 2Di, 2Ei, 3Ai, 3Bi and 3Ci). All five genes are expressed in broad overlapping domains in the animal hemisphere, but none are expressed at the animal pole, the prospective far anterior region. Expression is detected exclusively in the ectodermal, animal hemisphere precursors, not in the vegetal endomesodermal precursors at pregastrula stages. During gastrulation, the expression domains of most of the ligands become more spatially restricted, but generally they retain their relative spacing and order along the newly forming A/P axis of the ectoderm (Figs 2 and 3). As has been described in other species, many Wnt ligands are expressed around the forming blastopore; *wnt1* and *wnt4* (Fig 2Cii and 2Dii) are both expressed around the inside of the blastopore lip at the boundary of ectoderm and endomesoderm, whereas *wnt3*, *wnt6*, and *wnt16* (Fig 2Bii, 2Eii and 2Fii) are expressed further anteriorly in the ectoderm at the exterior edge of the blastopore lip. The remaining expression domains are located further anteriorly in the ectoderm; *wnt8*, *wnt2*, *wnt7*, and *wnt5* are all expressed anterior to *wnt3* in broadly overlapping domains (Fig 3Aii, 3Bii, 3Dii and 3Eii). *Wnt11* has the most anterior limit of ectodermal expression at this stage, but is still restricted from the most apical region (Fig 3Cii). As the embryos elongate after gastrulation, between 36 and 48 h of development, expression of the ligands becomes far more spatially restricted along the A/P axis. Most ligands remain expressed exclusively in the ectoderm with a few exceptions. Two of the ligands are expressed in the two posterior coeloms: *wntA* (Fig 3Fiv and 3Fv) and *wnt9* (Fig 3Giii–3Gv), and others are expressed in the endoderm: *wnt3* into the posterior endoderm (Fig 2Biii), *wnt9* in dorsal, anterior endoderm, around the forming gill slits (Fig 3Giii–3Gv), *wnt16* in the ventral posterior endoderm (Fig 2Fiii), and *wnt5* at low levels throughout the endoderm at 48 h of development, which later refines to the forming gill slits (Fig 3Eiv and 3Ev). *wnt1*, *3*, *4*, and *6* (Fig 2Biii, 2Ciii, 2Diii and 2Eiii) all retain their early expression around the blastopore. However, *wnt1*, *4*, *6*, and *16* develop additional expression domains around the anterior metasome (Fig 2Civ, 2Diii, 2Eiii and 2Fiv). *wnt8*, *2*, *11*, *7*, and *5* all begin to refine their single expression domains to the boundary between the prosome and metasome following gastrulation (Fig 3Aiii, 3Aiv, 3Biii, 3Biv, 3Ciii, 3Civ, 3Diii, 3Div, 3Eiii and 3Eiv), and by day three of development, some of these expression domains are sharply localized in narrow regions, such as in the case of *wnt8*, *2*, *and 7* (Fig 3Av, 3Bv and 3Dv). *wnt7* is not expressed as a single circumferential domain, and is down-regulated along the ventral midline by 48 h of development (Fig 3Div), and up-regulated in a dorsal spot at the base of the proboscis (Fig 3Dv).

By day three of development, all ligands with the exception of *wntA* and *wnt10* are detected in one or more of three ectodermal domains of expression; the base of the prosome, the boundary of the metasome and trunk at the developing first gill slit, and in the posterior ectoderm.

In summary, the zygotic expression of the Wnt ligands begins at the midblastula stage in the animal hemisphere, close to the boundary with the vegetal hemisphere. Throughout gastrulation, ligands are expressed broadly in the ectoderm from the blastopore to the more anterior regions of the ectoderm, but are always excluded from the most apical/anterior regions. By later developmental stages, this expression largely refines to spatially restricted domains marking a region in the posterior ectoderm, over the developing first gill slit, at the boundary between the trunk and collar, and finally at the boundary between the developing proboscis and the collar. Expression is also detected in the posterior endoderm and mesoderm.

### Wnt agonists and antagonists

An increasing number of modifiers of the cWnt pathway have been described in vertebrates [2,63]. Many of these genes encode secreted proteins, which act on the pathway by a variety of means and can act to both potentiate and inhibit cWnt signaling. Comparative studies on the expression and developmental roles of these proteins are far less extensive than studies on the Wnt ligands themselves. We have isolated and determined the expression pattern of several Wnt modifiers in *S*. *kowalevskii* and compared their expression and function to those reported in other animals in order to identify possible evolutionarily conserved roles of these pathway modifiers. Three antagonists are described below, and the other modifiers are described in S1 Text and S3 Fig.

#### Sfrps

**Fig 4:**
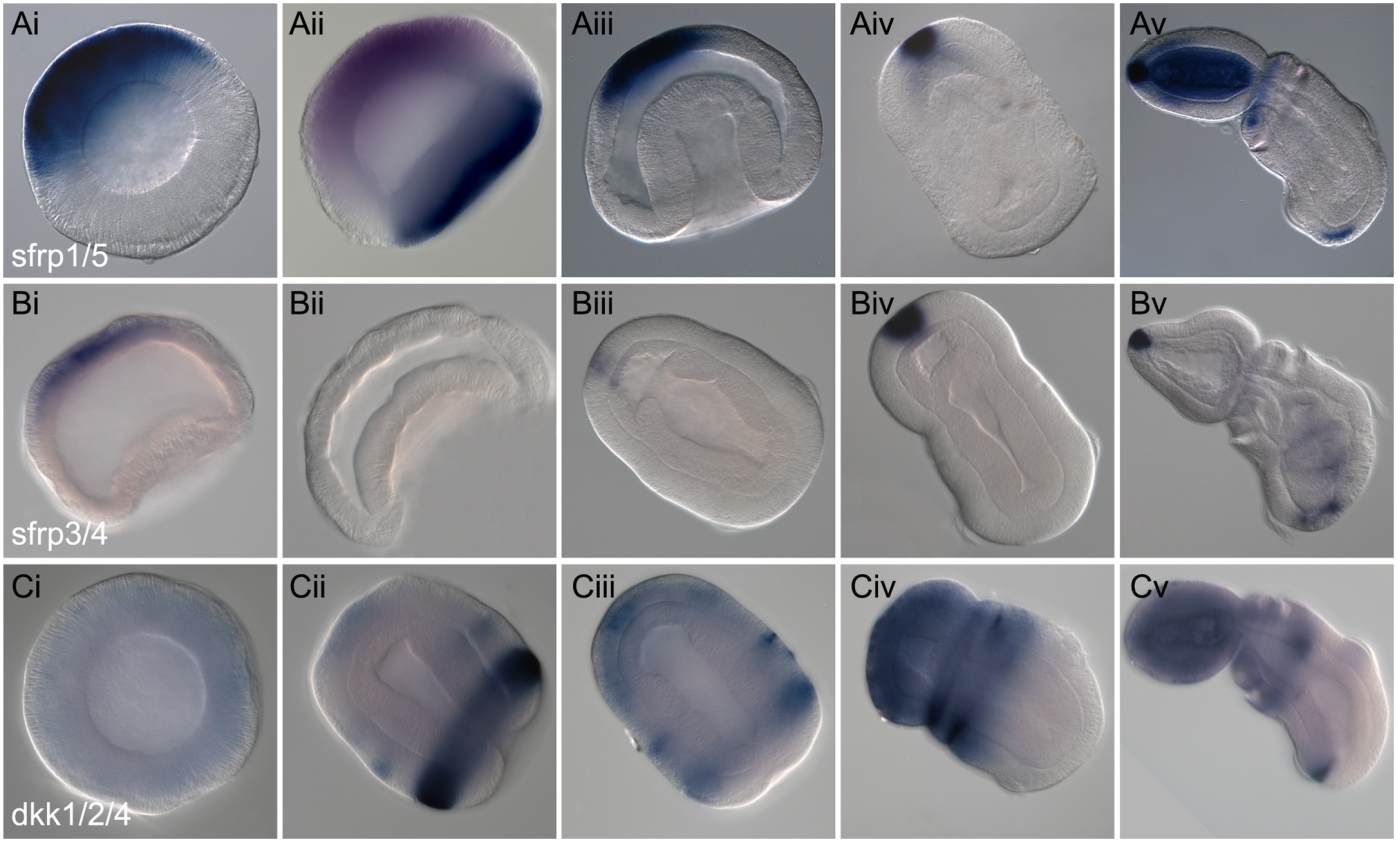
Expression of Wnt antagonists. Whole mount in situ hybridization of Wnt modifier genes. All data are presented as optical sagittal or frontal sections following clearing in Murray Clear. Developmental staging is from blastula stage to 72 h of development. All embryos are oriented with anterior, or animal (in the case of blastula), to the top left of the panel and posterior, or vegetal, to the bottom right of the panel. Right column, ventral is to the bottom left. Unless otherwise noted, expression is ectodermal. (A), Expression of *sfrp1/5* at blastula (Ai), coexpressed with the vegetal marker *foxA* (in blue) at late blastula stage (Aii), at midgastrula stage (Aiii), at 48 h in frontal section dorsal view (Aiv), and at 72 h of development side view. (B), Expression of *sfrp3/4* at late blastula stage (Bi), at early gastrula stage (Bii), at 48 h frontal view (Biii), at 60 hrs side view (Biv), and at 72 h of development (Bv). (C), Expression of *dkk1/2/4* at blastula stage (Ci), at late gastrula stage (Cii), at 36 h (Ciii), at 48 h, side view (Civ), and at 72 h of development in side view (Cv).

Members of the Sfrp family of Wnt inhibitors are modified receptors without the intracellular and transmembrane domains. They interact with both the Wnt ligands and the Fz receptors to block signaling and thus are not specific to the canonical pathway [63]. Sfrp-related proteins have been reported from sponges [4,64] and in many lophotrochozoan and ecdysozoan lineages, indicating their prebilaterian antiquity [65]. There are two main families of Sfrps: Sfrp1/5 and Sfrp3/4. Although Sfrps have diversified in the vertebrate lineage, we have cloned only one representative of each of the two groups in *S*. *kowalevskii* (S1D Fig), and survey of the current genomic assembly suggests this represents the entire complement. Both *S*. *kowalevskii* genes, *sfrp1/5* and *sfrp3/4*, are expressed in similar domains throughout early development (Fig 4A and 4B). Broad expression in the animal hemisphere is initiated at midblastula stages (Fig 4Ai, 4Aii and 4Bi) and then this domain becomes restricted to the more anterior ectoderm during gastrulation (Fig 4Aiii and 4Bii), and finally to the very anterior region in later stages (Fig 4Aiv, 4Av, 4Biii–4Bv), coincident with the long cilia of the apical organ of the hatched juvenile. This domain of expression is reminiscent of the distribution of mouse *sfrp1* and the closely related zebrafish *tlc* gene during the development of the vertebrate neural tube [26,66,67]. In juvenile stages, a small domain of expression of both genes is also detected in the far posterior ectoderm (Fig 4Av and 4Bv). Functional studies on sfrp1 and tlc have demonstrated the importance of this class of Sfrps in protecting the anterior nervous system from the posteriorizing effects of Wnts [26,68]. In addition to its ectodermal domain, a second domain of *sfrp1/5* is initiated in the anterior mesoderm after gastrulation and following the specification of the anterior coelom of the proboscis (Fig 4Aiv and 4Av) [48]. Vertebrate *frzb* from the Sfrp3/4 class is also expressed anteriorly, but in the prechordal plate mesoderm of the head organizer [21,69,70]. It, too, has a demonstrated role in Wnt antagonism and head development [21,71]. Sea urchins also express *sfrp1/5* in the anterior embryonic ectoderm and a role of cWnt in patterning this territory has been established through a series of experiments [34,72]. Expression of *sfrp1/5* has recently been described in the indirect-developing species *Ptychodera flava* in the anterior ectoderm [73]. Based on the anterior locations of these domains, the data are consistent with hemichordate Sfrps being involved in establishment of a cWnt activity gradient, and a functional role of cWnt signaling in the establishment and patterning of the ectodermal A/P axis, and is tested later experimentally.

#### Dkk1/2/4

The role of the Dkk1/2/4 class of genes is well-characterized in vertebrates and is represented by the following three genes: *dkk1*, *2*, and *4*. Of these paralogues, *dkk1* is the most comprehensively characterized functionally [25,63], but all three act as cWnt antagonists (although in some cases as agonists) by interacting with the Fz coreceptors, LRP5/6, and Kremen [25,74,75]. Dkk1 is secreted from the head organizer mesoderm during gastrulation, and its overexpression—in conjunction with BMP antagonists—induces a second head in *Xenopus laevis*. Injection of anti-Dkk1 antibodies results in microcephalic embryos [25]. In sea urchins, *dkk1/2/4* is expressed in the anterior ectoderm and has a demonstrated role in patterning the larval neurectodermal territory in a manner mechanistically similar to vertebrate anterior neural patterning [34]. In two groups of cnidarians, *dkk1/2/4* is localized to the aboral end of the planula [76] and to the foot of the polyp, opposite to the Wnt positive domain [77]. This expression is suggestive of a similar role of Dkk in cWnt antagonism during late axial patterning in these cnidarians, although functional experiments are still required to test the predictions.

In *S*. *kowalevskii*, we have isolated a single *dkk1/2/4* gene (S2A Fig). Expression is not detectable at the late blastula stage (Fig 4Ci) but begins during gastrulation, most prominently in the ciliated band, and at lower levels in an anterior ectodermal ring and apical spot (Fig 4Cii). By 36 h of development, the expression in the ciliated band has decreased and the anterior spot of expression expands to the entire prospective proboscis ectoderm (Fig 4Ciii). By 48 h, the expression is broadly localized to the anterior ectoderm, that is, to anterior trunk, collar, and proboscis ectoderm (Fig 4Civ). It is no longer detectable in the ciliated band. By 72 h, the expression remains localized in the anterior ectoderm extending posteriorly to the anterior trunk with expression in the dorsal most part of the developing gill slits. An additional ventral ectodermal domain is activated immediately posterior to the ciliated band (Fig 4Cv). In summary, *dkk1/2/4* exhibits quite dynamic expression during early development. Similar to the other antagonists, it has a broad anterior localization throughout early development, lessening posteriorly. However, additional posterior domains are present indicating the possibility of functions in early A/P axis establishment followed by subsequent roles in axial patterning.

### Fz receptors

We cloned four representatives of the Fz receptor family characterized by an extracellular domain, including a signal peptide, and a cysteine-rich Wnt-binding domain, seven transmembrane domains, and a cytoplasmic tail [78]. A genomic survey of the current genome assembly identified only four Fz genes. Previous studies have described four ancestral Fz subgroups: Fz1/2/7, Fz4, Fz5/8, and Fz9/10 [62]. The four Fzs from hemichordates exhibit robust orthology to these subgroups already present in cnidarians (S1B Fig) and build further support to the hypothesis that four Fzs are ancestral to the eumetazoan lineage [79]. Similar to the findings from echinoderms, which are a sister group to hemichordates, we were not able to isolate a member of the Fz3/6 group, which is present in vertebrates but absent from cnidarians and sea urchins. This supports the hypothesis that the group arose by duplication during chordate evolution [62].

**Fig 5:**
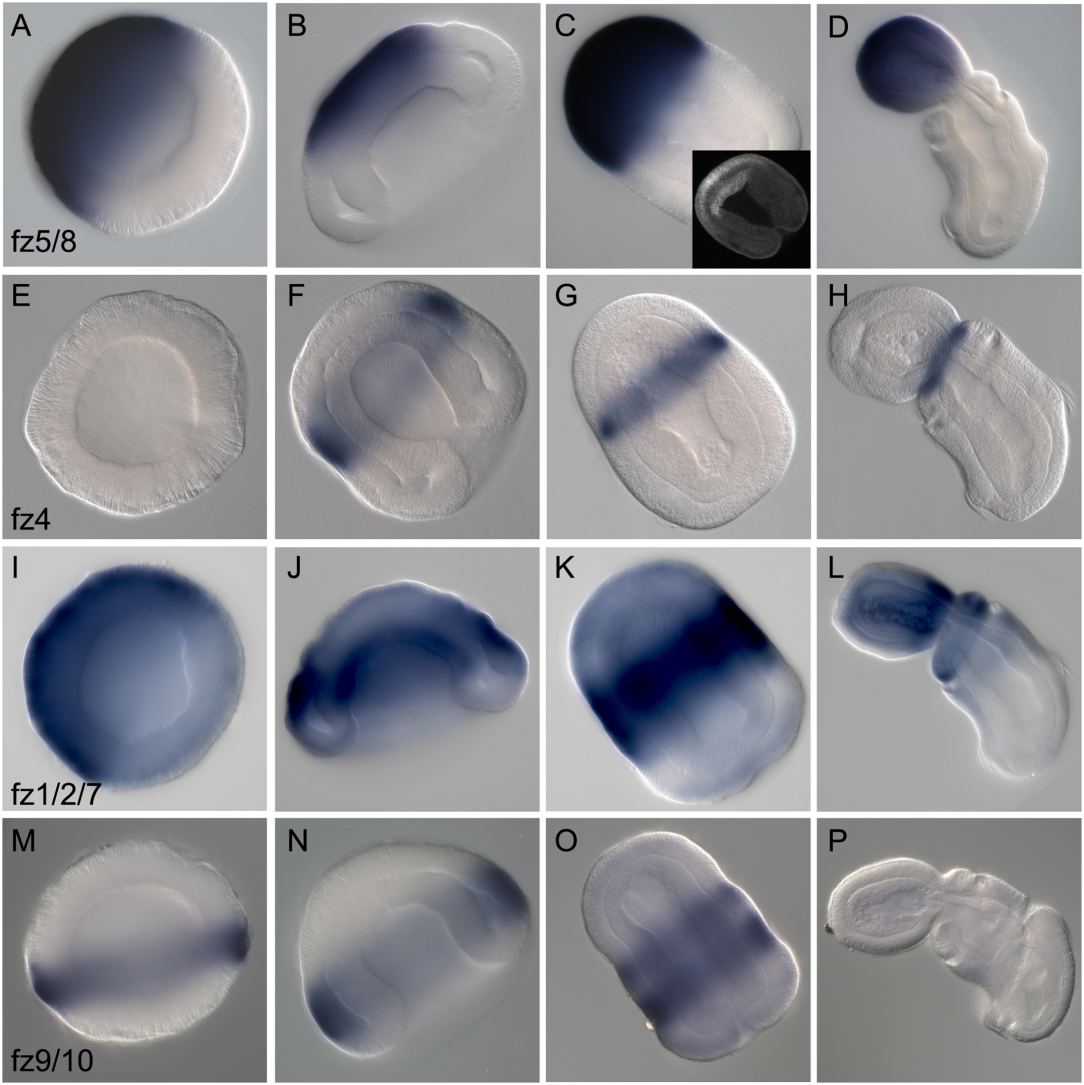
Expression of Fz receptors. Whole mount in situ hybridization of Fz genes. All data are presented as optical sagittal or frontal sections following clearing in Murray Clear. Developmental staging is from blastula to 72 h of development. All embryos are oriented with anterior, or animal (in the case of blastula), to the top left of the panel and posterior, or vegetal, to the bottom right of the panel. Right column, ventral is to the bottom left. (A-D), Expression of *fz5/8* at blastula stage (A), at early gastrula stage (B), at 36 h of development, with bottom right inset showing optical section of a late gastrula stage embryo labelled with a fluorescent probe showing expression in the anterior endomesoderm (C), and at 72 h of development in side view (D). (E-H), Expression of *fz4* at blastula stage (E), midgastrula stage (F), at 36 h of development in dorsal view (G), and at 60 h of development in side view (H). (I-L), Expression of *fz1/2/7* at blastula stage (I), at early gastrula stage (J), at 48 h of development (K), and at 60 h of development in side view (L). (M-P), Expression of *fz9/10* at blastula stage (M), at midgastrula stage (N), at 48 h of development (O), and at 60 h of development in side view (P). Fz, frizzled.

Partial expression profiles of *fz5/8* in *S*. *kowalevskii* and early embryonic and larval expression in *P*. *flava* have been previously described in the anterior of both embryos [48,73]. By RT-PCR, two Fzs, *fz5/8* and *fz1/2/7* are detected at high levels maternally in newly fertilized oocytes and early cleavage stages (Fig 1). By late blastula/early gastrula and subsequent developmental stages, all Fzs are expressed. Their expression, like the Wnt ligands, is also highly regionalized along the A/P axis during all developmental stages, suggesting that they play region-specific roles during the patterning of the embryonic A/P axis. The most anteriorly expressed of the receptors is *fz5/8*, detected midway through blastula from the animal pole throughout most of the animal hemisphere, but excluded from the most vegetal region of that hemisphere, the region fated as posterior ectoderm (Fig 5A). As gastrulation begins, the expression is increasingly restricted to the most anterior ectoderm. As the vegetal hemisphere invaginates and contacts the anterior ectoderm midway through gastrulation, *fz5/8* expression is initiated in the anterior endomesoderm that is fated to become the proboscis/prosome mesoderm, at the same A/P level as the ectodermal expression (Fig 5C). As gastrulation continues, the expression is now clearly restricted in the anterior ectoderm of the prospective prosome, and continues in this domain [48] throughout all stages examined. This expression resembles the early expression of *fz5/8* in both *P*. *flava*, sea urchin, and the anterior localization of *fz8* during mouse development [72,73,80]. Short interfering RNA (siRNA) knockdown of *fz5/8* resulted in abnormal patterning of the anterior ectoderm with the expansion of apical markers at the expense of more posterior proboscis markers, demonstrating the importance of Wnt signaling for the posteriorization of the anterior ectodermal territory [48].

*fz4* is expressed in a highly restricted domain from the onset of zygotic expression during gastrulation (Fig 5F). Expression is uniquely ectodermal and is localized to a narrow ring in the more rostral region of the anterior ectoderm. This domain refines to a narrow ring at the boundary between the prosome and mesosome, at the posterior boundary of the expression limit of *fz5/8* (Fig 5G and 5H). This to some extent resembles the expression of *fz4* in chick and mouse, where it is localized in the diencephalon at the boundary with the telencephalon [80]. *fz1/2/7* has a much broader domain of expression; it is first detected at blastula stage throughout the animal hemisphere, more broadly than that of *fz5/8* (Fig 5I). By the beginning of gastrulation, its expression has been largely down-regulated in the anterior ectoderm and around the blastopore, but remains in the midectoderm (Fig 5J). Expression is also detected in the forming mesendoderm. By 36 h after fertilization, transcripts are detected in overlapping domains with both *fz4* and *fz5/8* in the anterior domain, with expression mainly in the prospective mesosome, expanding down into the prospective trunk, but not past the ciliated band into the blastoporal area (Fig 5K). By day three of development, most transcripts are detected in the mesosome and at the base of the proboscis, with only weak expression in the anterior trunk. At this late stage, expression is now visible in the anterior mesoderm of the proboscis (Fig 5L). *fz9/10* expression begins at blastula in the most vegetal portion of the animal hemisphere (Fig 5M). At gastrulation, expression remains in a similar domain in the posterior ectoderm, just anterior to the forming ciliated band (Fig 5N), and by 36 h of development, the expression domain has expanded anteriorly to the entire trunk and part of the collar, overlapping extensively with the expression domain of *fz1/2/7* (Fig 5O). By day three of development, expression is no longer detectable.

The interactions of Fzs and Wnts is poorly understood, and it is proposed that the overlapping expression of many of the vertebrate Fzs leads to functional redundancy [81,82]. In *S*. *kowalevskii*, Fz expression is highly regionalized along the A/P axis and suggests that the different receptors are responsible for patterning different regions of the axis. Notably, none of the receptors are expressed around the blastopore, where many of the ligands are localized. The functional significance of this observation is explored in later experiments.

### Functional experiments

**Fig 6:**
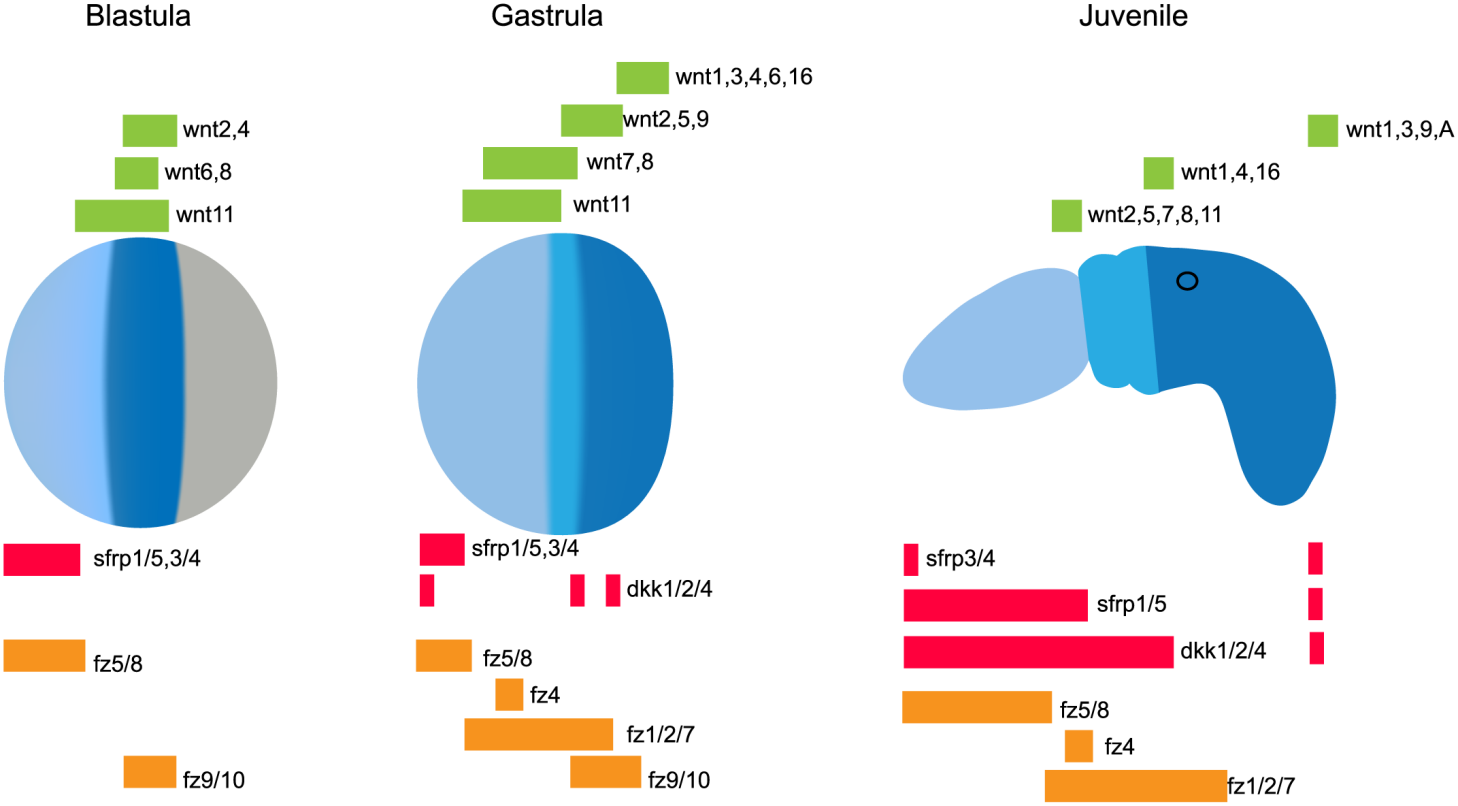
Summary of Wnts, Wnt antagonists, and Fzs receptor expression. Wnts are expressed in nested domains posteriorly, whereas *sfrps* are expressed in the anteriormost ectoderm at the blastula and gastrula stages. *dkk1/2/4* is expressed in three discrete domains of gastrula ectoderm. At juvenile stages, Sfrps are expressed in the very anterior ectoderm (apical tuft), and *sfrp1/5* is also expressed in the entire proboscis mesoderm, whereas *dkk1/2/4* is broadly expressed in the anterior ectoderm. Wnts are expressed in three discrete ectodermal domains: the base of the proboscis, the anterior trunk (over the first gill slit), and the posterior-most ectoderm. In addition, *wnt9* and *wntA* are expressed in posterior internal tissues. Fz genes are expressed in nested domains along the ectoderm. Territories are color coded: endomesoderm (grey), posterior ectoderm (dark blue), intermediate ectoderm (medium blue) and anterior ectoderm (light blue). Fz, frizzled; Sfrp, secreted frizzled-related protein.

The expression of Wnt ligands and their antagonists is strongly localized along the developing A/P axis of *S*. *kowalevskii*, and their relative domains are similar in location to their orthologues during vertebrate development; ligands are expressed posteriorly and antagonists are expressed anteriorly, with Fz receptors expressed in staggered domains (Fig 6). We carried out a series of experiments to test whether the cWnt pathway is involved in the early specification of the hemichordate A/P axis.

### Overactivation of the cWnt pathway leads to anterior truncation and ectoderm posteriorization

#### GSK3β inhibition

**Fig 7:**
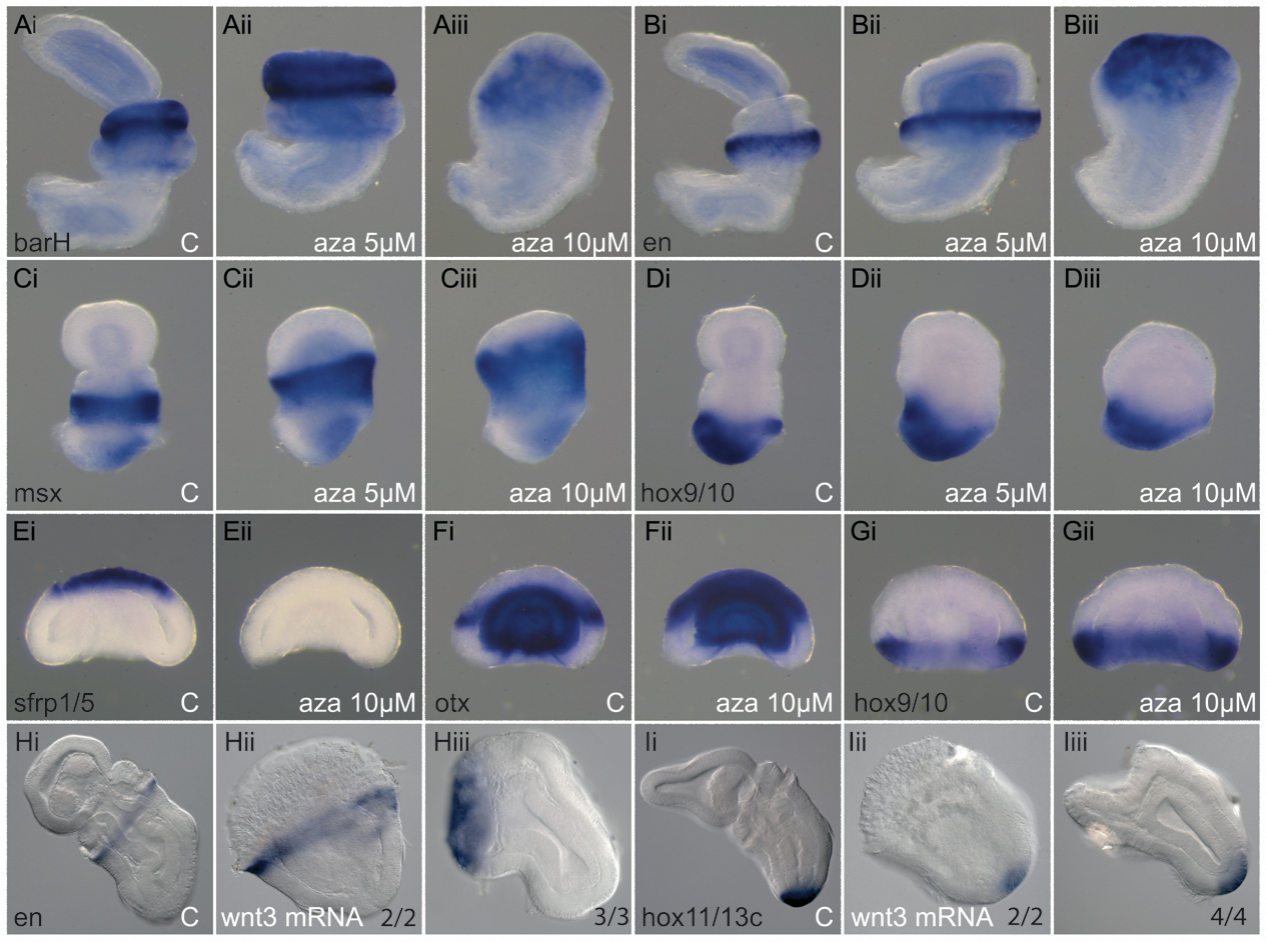
Activation of the cWnt pathway leads to anterior truncation. (A-D), Treatment of embryos with the GSK3β inhibitor 1-azakenpaullone leads to a loss of proboscis at 5 μM (Aii, Bii, Cii, Dii) and to a loss of both proboscis and collar at 10 μM (Aiii, Biii, Ciii, Diii). DMSO-treated control embryos (Ai, Bi, Ci, Di). In situ hybridization for ectodermal markers of the anterior collar *barH* (A), anterior trunk *engrailed* (B), trunk *msx* (C), and posterior trunk *hox9/10* (D). Embryos at two and a half (C-D) and five (A-B) days of development. Earlier sampling at gastrula stage shows no morphological change but significant transformation of markers *sfrp1/5* (Eii) and *otx* (Fii), but no change in *hox9/10* (Gii) at 10 μM 1-azakenpaullone. DMSO control embryos (Ei, Fi, Gi). Anterior to the top, ventral to the left. (H-I), Overexpression of *Wnt3* by mRNA injection produces virtually identical phenotypes: loss of proboscis (Hii and Iii) or loss of proboscis and collar (Hiii and Iiii), depending on the strength of the phenotype. In situ hybridization for ectodermal markers of the anterior trunk *engrailed* (H) and posterior trunk *hox11/13c* (I) at three days of development (numbers indicate embryos with the displayed phenotyped over the number of analyzed embryos). Anterior to the top left, ventral to bottom left. C, control embryo; cWnt, canonical Wnt; DMSO, dimethyl sulfoxide; GSK3β, glycogen synthase kinase 3 beta.

To overactivate the cWnt pathway at specific developmental stages, we used an inhibitor of GSK3β, 1-azakenpaullone [54], which is an intracellular component of the cWnt signaling pathway. In the absence of Wnt activation, GSK3β targets β-catenin for degradation, but upon cWnt activation, GSK3β is inhibited and results in accumulation of β-catenin and activation of downstream targets. We have previously shown that treating *Saccoglossus* embryos with this inhibitor during cleavage stages is sufficient to convert all embryonic cells into endomesoderm [15]. To directly address Wnt involvement in A/P axis patterning, we treated embryos from early blastula to early gastrula, a treatment that does not elicit ectopic endomesoderm formation [15]. The resulting embryos exhibited severe anterior truncations with the severity of phenotype positively correlated with the concentration of inhibitor used (Fig 7Ai–7Diii). At the 5 μM level, the entire proboscis/prosome was absent, and at 10 μM, in addition to the proboscis, the collar/mesosome was also missing. The remaining trunk, however, seemed normal in its morphology. These morphological observations are confirmed by molecular markers of the A/P axis (Fig 7) [47]. At the lower inhibitor concentration, the anterior limit of the collar marker *barh* was displaced to the anterior of the embryos (Fig 7Aii), and at the highest concentration, most of the expression was lost (Fig 7Aiii). Similarly, the rostral expression limit of the anterior trunk marker *en* (Fig 7Bii) was displaced anteriorly at low concentrations of the inhibitor (Fig 7Bii), and at the highest concentration was expressed at the far anterior limit of the embryo (Fig 7Biii), confirming the morphological interpretation of anterior truncation. Similarly, the trunk marker *msx* (Fig 7Ci) was expanded anteriorly following treatments (Fig 7Cii and 7Ciii). Expansion of the most posterior territories was not observed following inhibitor treatment, as demonstrated by the expression of *hox9/10* (Fig 7Di), which was largely unaffected (Fig 7Di and 7Dii). These data suggest that the regions of the A/P axis have differential sensitivity to cWnt signaling, and we investigated this further in later experiments. Molecular marker analysis suggests that anterior parts of the embryo are missing because they are re-specified into more posterior tissue. This is more evident when expression is analyzed at early gastrula stages, at a time when control and treated embryos are morphologically indistinguishable (Fig 7Ei–7Gii). We also assayed for effects on the endomesoderm following 1-azakenpaullone treatments by examining expression of *foxA* and *caudal*, which normally mark different domains of the endoderm. Following treatment, expression of both genes expands to the far anterior endomesoderm (S4 Fig).

#### Overexpression of *wnt3*

*wnt3* overexpression by mRNA injection resulted in very similar phenotypes to 1-azakenpaullone treatments. The range of phenotypes closely resembled those obtained from 5 and 10 μM treatments; anterior structures are truncated to various degrees resulting in loss of the proboscis/prosome in the mildest case (Fig 7Hii and 7Iii) and loss of the entire proboscis and collar (Fig 7Hiii and 7Iiii) in the most severe case. The posterior marker *hox11/13c* was largely unaffected, remaining around the most posterior region of the embryo (Fig 7I), whereas *en* expression in the anterior trunk domain shifted anteriorly (Fig 7Hii and 7Hiii). Treatment of embryos with recombinant murine Wnt3a protein led to very similar phenotypes (S5 Fig).

In summary, strategies to increase Wnt signaling and β-catenin levels in embryos had similar effects; the loss of anterior structures with relatively normal trunk formation.

### Timing of A/P Wnt sensitivity during early development

To test for the timing of Wnt activity during early A/P patterning, we further developed the 1-azakenpaullone experiments. Concurrent treatments were carried out by adding the inhibitor every 2 h, with the first beginning at early blastula (11 hpf), and the treatment ending at 64 hpf (S6 Fig). Embryos were cultured at 20°C and fixed at 96 hpf when all the major body divisions had formed, and the A/P and dorsoventral (D/V) axes were clearly morphologically differentiated. Graded phenotypes were observed and correlated with the treatment initiation time; the more severe phenotypes resulted from earlier and longer treatments (S6I Fig). The first treatment initiated at 11 h led to the most dramatic phenotype that we have observed. The embryos exhibited major morphological defects; all anterior markers down to anterior trunk failed to express, even the anterior trunk marker *en* (S6ii Fig), but *msx*—normally a broad trunk marker—was expressed up to the most anterior region of the embryo (S6Gii Fig). *hox9/10*, a posterior trunk marker, was expressed almost up to the anterior of the treated embryos (S6Hii Fig). Later treatments produced weaker phenotypes with a gradual restoration of molecular marker expression from posterior to anterior. *hox9/10* expression was restricted posteriorly for treatments starting at 13 hpf or later, and *msx* expression did not expand anteriorly for treatments starting at 17 hpf or later (S6G and S6H Fig). These experiments suggest that the ectoderm is most sensitive to cWnt activation during the blastula and gastrula stages. It is the period when Wnt genes are expressed broadly in the ectoderm (Fig 6).

### A biphasic sensitivity of posterior markers to cWnt activation

**Fig 8:**
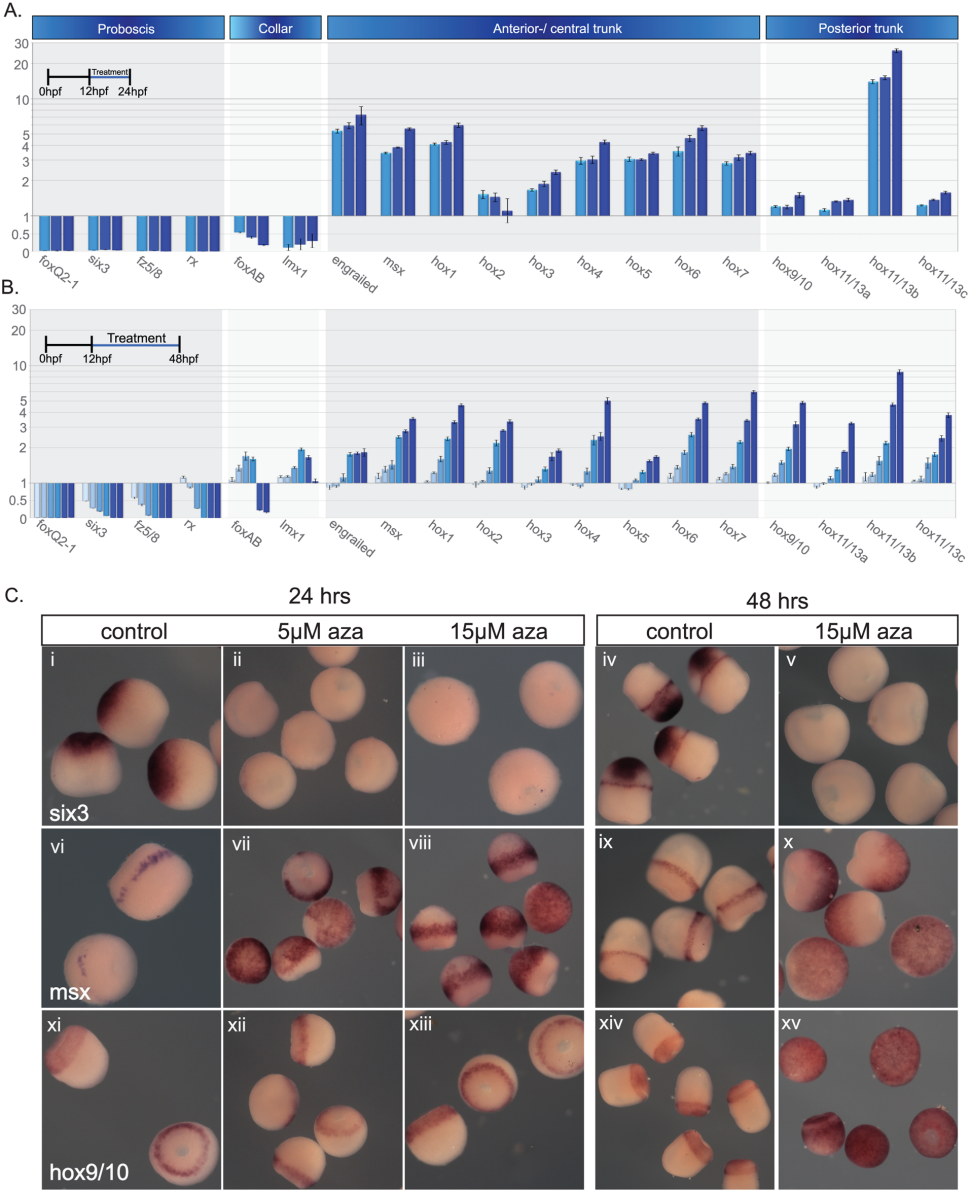
Activation of cWnt has differing effects along the A/P axis. (A,B), qPCR results for embryo treatments with 1-azakenpaullone at a range of concentrations. (A), Embryos treated from 12 hpf to 24 h at 5, 10, and 15 μM. (B), Embryos treated from 12 h to 48 h at 0.1, 0.5, 1, 5, 10, and 15 μM. Light blue represents the lowest concentration and dark blue the highest in both A and B. (C), The same treatments as described in (A) and (B), but embryos fixed and examined by in situ hybridization for a selection of axial markers. Raw data files for qPCR (S1 and S2 Data). A/P, anteroposterior; aza, 1-azakenpaullone; cWnt, canonical Wnt; hpf, hours postfertilization; qPCR, quantitative PCR.

We have further analyzed the above experiment by examining gene expression at 21 hpf (onset of gastrulation), when little morphogenesis has occurred (S6B–S6D Fig). A similar gradation in phenotypes was observed for the anterior markers *sfrp1/5* and *six3*. Interestingly, expression of the posterior marker *hox9/10* was unchanged even for the earliest treatment starting at 11 hpf, while we observed an ectopic expression anteriorly when its expression was analyzed at 96 hpf (compare S6Dii and S6Hii Fig). To better understand this discrepancy, we treated embryos with a range of 1-azakenpaullone concentrations starting at early blastula (12 hpf) and analyzed A/P ectodermal markers expression using quantitative PCR (qPCR) at early gastrula stages (24 hpf; Fig 8A). This restricted the treatment to early establishment of the A/P axis. The second treatment again began at 12 h but was extended to 48 h into early embryo elongation from 24 to 48 h, after the early specification of the A/P axis (48 hpf; Fig 8B). For both treatments, proboscis/prosome markers were strongly repressed (Fig 8A and 8B). Collar markers were initially strongly down-regulated, whereas their later expression was either moderately activated or repressed depending on the inhibitor concentration (Fig 8A and 8B). This observation suggests that anterior suppression regulates fates down to the collar and its exact position varies according to the concentration of 1-azakenpaullone. All anterior/central trunk markers are activated in a dose-dependent manner at both stages. Early expression of posterior Hox genes, during the initial establishment of the posterior territory, is insensitive to cWnt activation, with the exception of *hox11/13b*. By contrast, the same genes are strongly activated when their expression is analyzed at 48 hpf. We confirmed this global gene expression analysis by in situ hybridization of selected markers (Fig 8C). The anterior gene *six3* is not detected following either treatment. The expression of the trunk marker *msx* was expanded anteriorly for both treatments while the posterior marker *hox9/10* was only ectopically activated when analyzed at 48 hpf during embryo elongation following gastrulation.

Overall, the results from this section indicate that A/P markers can be organized into three groups according to their sensitivity to cWnt activation: (1) anterior genes are repressed (proboscis) or activated or repressed depending on concentration (collar), (2) intermediate genes (anterior/central trunk) are activated, and (3) posterior genes (posterior trunk) are initially insensitive before being activated following gastrulation during embryo elongation.

### cWnt pathway activation posteriorizes isolated ectoderm

**Fig 9:**
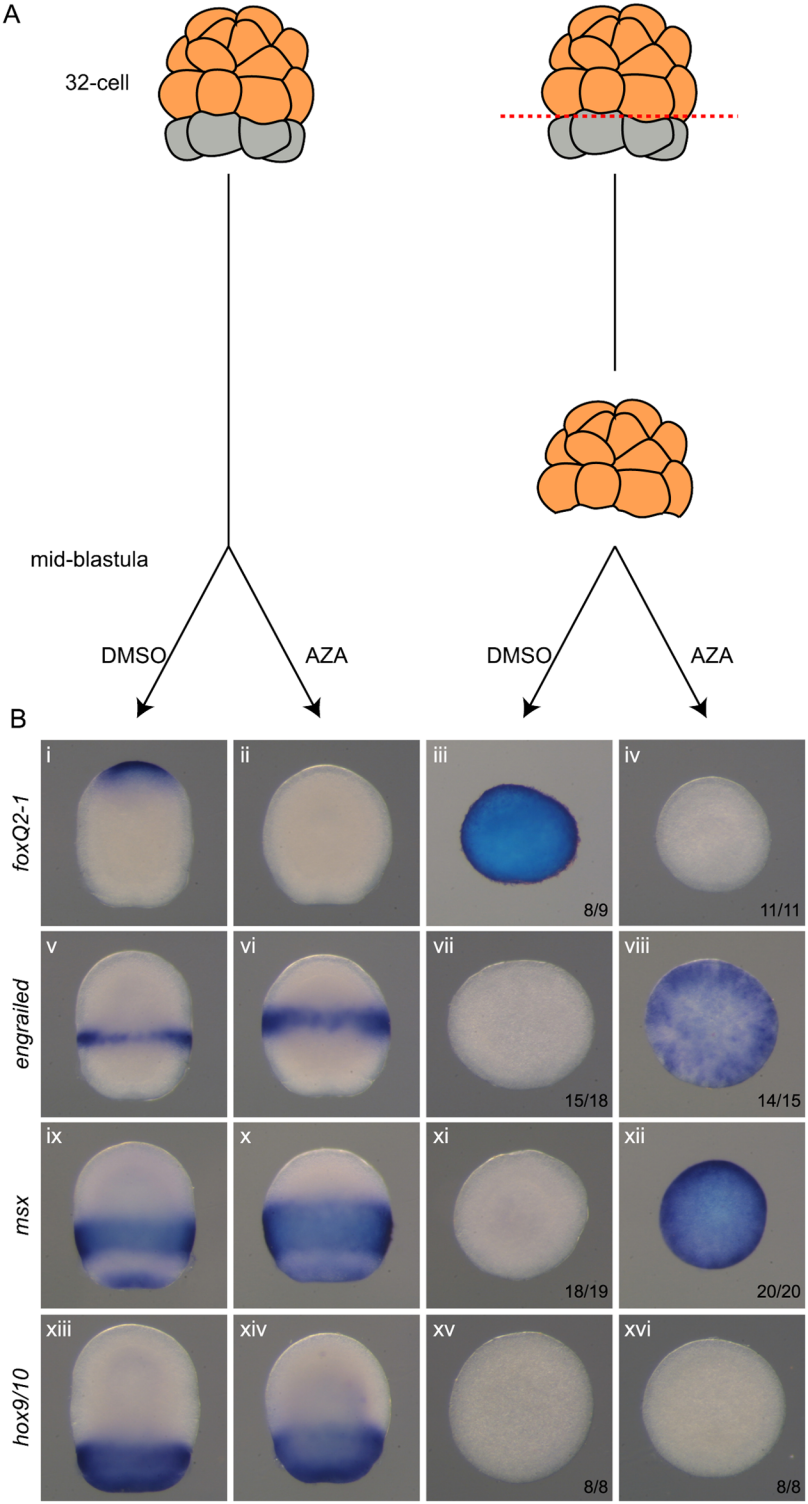
Activation of the cWnt pathway is sufficient to posteriorize naive ectoderm. (A), The experimental scheme is as follows: ectodermal precursors (orange) were separated from endomesoderm precursors (grey) at the 32-cell stage and cultured until midblastula stages (12 h), when they were treated with 10 μM of 1-azakenpaullone until fixation at 30 h. (B), Upon activation of the Wnt pathway, anterior fate of naive ectoderm is repressed, whereas more posterior identity is activated except for the most posterior one. In situ hybridization for ectodermal markers of the anterior ectoderm *foxQ2-1* (i-vi), anterior trunk *engrailed* (v-viii), trunk *msx* (ix-xii), and posterior trunk *hox9/10* (xiii-xvi). (i, v, ix, and xiii), DMSO-treated control embryos. (ii, vi, x and xiv), 1-azakenpaullone treated embryos. (iii, vii, xi and xv), DMSO-treated 32-cell explants. (iv, viii, xii and xvi), 1-azakenpaullone treated 32-cell explants. Embryos are shown with anterior to the top. Numbers indicate the number of explants whose expression corresponds to the picture over the number of explants analyzed. AZA, 1-azakenpaullone; cWnt, canonical Wnt; DMSO, dimethyl sulfoxide.

In hemichordates, we have shown that endomesoderm is the source of early signals posteriorizing the ectoderm that otherwise adopt an anterior character (Fig 9) [15]. We tested whether activation of the Wnt pathway was sufficient to posteriorize naive ectoderm explants that lack inputs from the endomesoderm. Embryos were cut at the 32-cell stage and the animal hemispheres cultured in isolation. They developed anterior fates demonstrated by the expression of the apical marker *foxQ2-1* throughout the explant (Fig 9Biii), and failed to express posterior transcriptional markers such as *en*, *msx*, and *hox9/10* (Fig 9Bvii, 9Bxi and 9Bxv). However, treating these explants with 1-azakenpaullone from midblastula stages (12 h of development) resulted in repression of the apical marker *foxQ2-1* (Fig 9Biv) and the ectopic activation of the more posterior markers, *msx*, and *en* (Fig 9Bvii and 9Bxii) throughout the ectoderm. However, we were unable to activate the most posterior marker *hox9/10* (Fig 9Bxvi). The expression of this marker was actually unchanged upon cWnt activation in whole embryos (Fig 9Bxiv), whereas expression of both *en* and *msx* was expanded (Fig 9Bvi and 9Bx). These results are entirely consistent from the 1-azakenpaulone treatments in intact embryos, and further suggest that there is a differential response to Wnt signaling along the A/P axis, with three distinct domains.

### Inhibition of cWnt anteriorizes the ectoderm with the exception of posterior ectoderm

**Fig 10:**
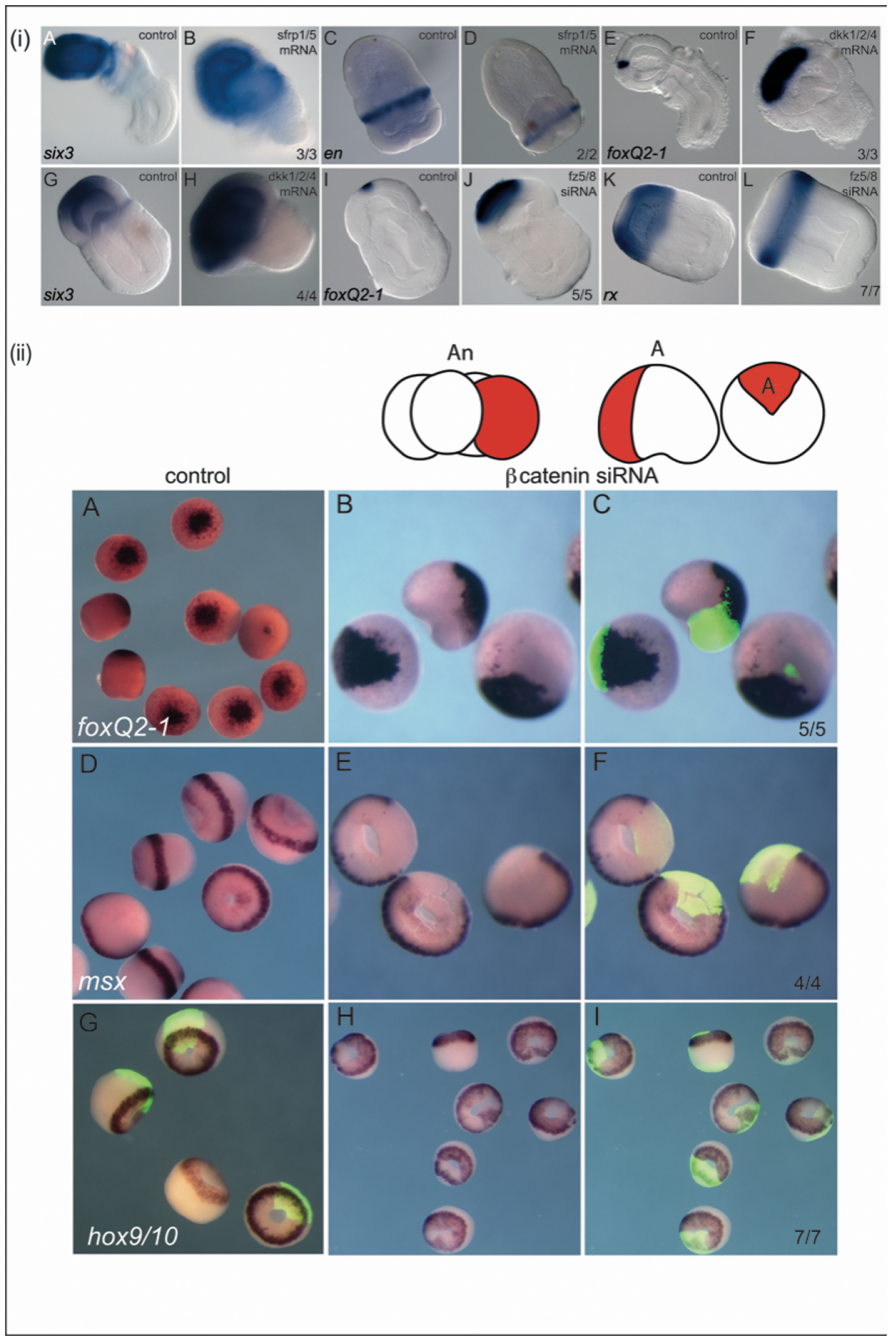
Blocking cWnt pathway anteriorizes the embryo. (i), Overexpression of *sfrp1/5* and *dkk1/2/4* produces embryos with enlarged proboscis and reduced trunk. Control embryos (A, C, E, G, I and K). Embryos injected with *sfrp1/5* mRNA (B and D), *dkk1/2/4* mRNA (F and H), and *fz5/8* siRNA (J and L). In situ hybridization for the most apical marker *foxQ2-1* (E, F, I and J), the proboscis markers *six3* (A, B, G and G) and *rx* (K and L), and the anterior trunk marker *engrailed* (C and D). 48 h embryos (C, D, G-L). Seventy-two h embryos (A, B, E and F). Anterior to the top left, ventral to the bottom left. Numbers indicate embryos with the displayed phenotyped over the number of analyzed embryos. (ii), Knock down of β-catenin extends anterior-most fate only into anterior ectoderm. siRNA against β-catenin was injected at 4-cell stage into single blastomeres with a rhodamine tracer (red cell in diagrams, which then develop into a full quadrant of the embryo at gastrula, also shown in the model in red). Expression by in situ hybridization at 28 h of the apical marker *foxQ2-1* (A-C), the midtrunk marker *msx* (D-F), and the posterior marker *hox9/10* (G-I). Control embryos are shown in (A,D and G), and G represents an injection control. Panels B, E, and H all show the expression of markers genes following injection of β-catenin siRNA. Panels C, F, and I also show the fluorescent tracer showing the lineage of the injected blastomeres at gastrulation. siRNA, short interfering RNA.

We carried out reciprocal experiments to test for the effects of reducing Wnt activity. We first injected capped mRNA of the Wnt antagonists *dkk1/2/4* and *sfrp1/5*. Both overexpressions led to very similar phenotypes; expansion of the anterior proboscis territory and reduction in the size of the trunk (Fig 10i, S1 and S2 Movies), which is the reciprocal phenotype from Wnt3 overexpression (Fig 7Hi–7Iiii). The phenotypes differed in that the proboscis took on a more bulbous appearance with a narrower trunk following *dkk1/2/4* injection (Fig 10iF and 10iH) when compared to *sfrp1/5*-injected embryos (Fig 10iB and 10iD). This may be due to the difference in specificity of the secreted antagonists as Dkk1/2/4 specifically antagonizes the canonical pathway by binding to Kremen/lrp, whereas Sfrp1/5 binds the Fz receptor and inhibits all Fz-mediated Wnt signaling. The molecular markers support the phenotypic transformations with both *foxQ2-1* and *six3* expanding in injected embryos (Fig 10iB, 10iF and 10iH), and the trunk marker *en* expressed further posteriorly with weaker expression when compared to controls following injection of *sfrp1/5* (Fig 10iD), consistent with down-regulation of this trunk marker. Previous data have revealed that knockdown by siRNA of *fz5/8* results in an anteriorized phenotype, as assayed by the expansion of the apical marker SkFGF-1; the effect is restricted to the proboscis matching the expression domain of *fz5/8* [48]. We further tested this phenotype by assaying the expression of another apical marker *foxQ2-1* and show a similar expansion (Fig 10iI and 10iJ). Conversely, the expression domain in experimental embryos of *rx*, a proboscis ectodermal marker normally excluded from the most apical domain, is restricted to a more posterior domain of expression (Fig 10iK and 10iL) [48], suggesting that a gradient of Wnt activity is involved in patterning the proboscis ectoderm.

The overexpression of Wnt antagonists results in a phenotype complementary to cWnt activation, but trunk fates cannot be repressed entirely. Because this could result from an incomplete blockade of cWnt activity, we performed additional experiments utilizing targeted injections of siRNAs designed to β-catenin. However, β-catenin is necessary and sufficient to specify endomesoderm, and injection of siRNA against β-catenin before the first cell cycle disrupts the establishment of the AV axis, resulting in fully animalized embryos that fail to gastrulate [15]. To avoid disrupting germ layer formation, β-catenin siRNA was injected into single blastomeres at the 4-cell stage. The first two cleavage planes occur along the AV axis and define the left/right and dorso-ventral axes, respectively [53]. Gastrulation and endomesoderm formation (revealed by *foxA* expression) were relatively normal if injection was delayed until the 4-cell stage (Figs 10ii and S7). Expression of *foxQ2-1*, which is normally restricted to the anterior-most ectoderm (Fig 10iiA), expanded posteriorly in descendants of blastomeres injected at the 2,4, and 8-cell stage (Fig 10iiB and 10iiC and S7 Fig). Notably, expression did not expand down to the presumptive posterior ectoderm, despite the presence of the siRNA in all ectodermal descendants in the injected quadrant (Fig 10iiC). Expression of the trunk marker *msx* was lost in all descendants of injected cells (Fig 10iiE and 10iiF), while the posterior marker *hox9/10* was activated even in the absence of cWnt signalling (Fig 10iiH and 10iiI).

**Fig 11:**
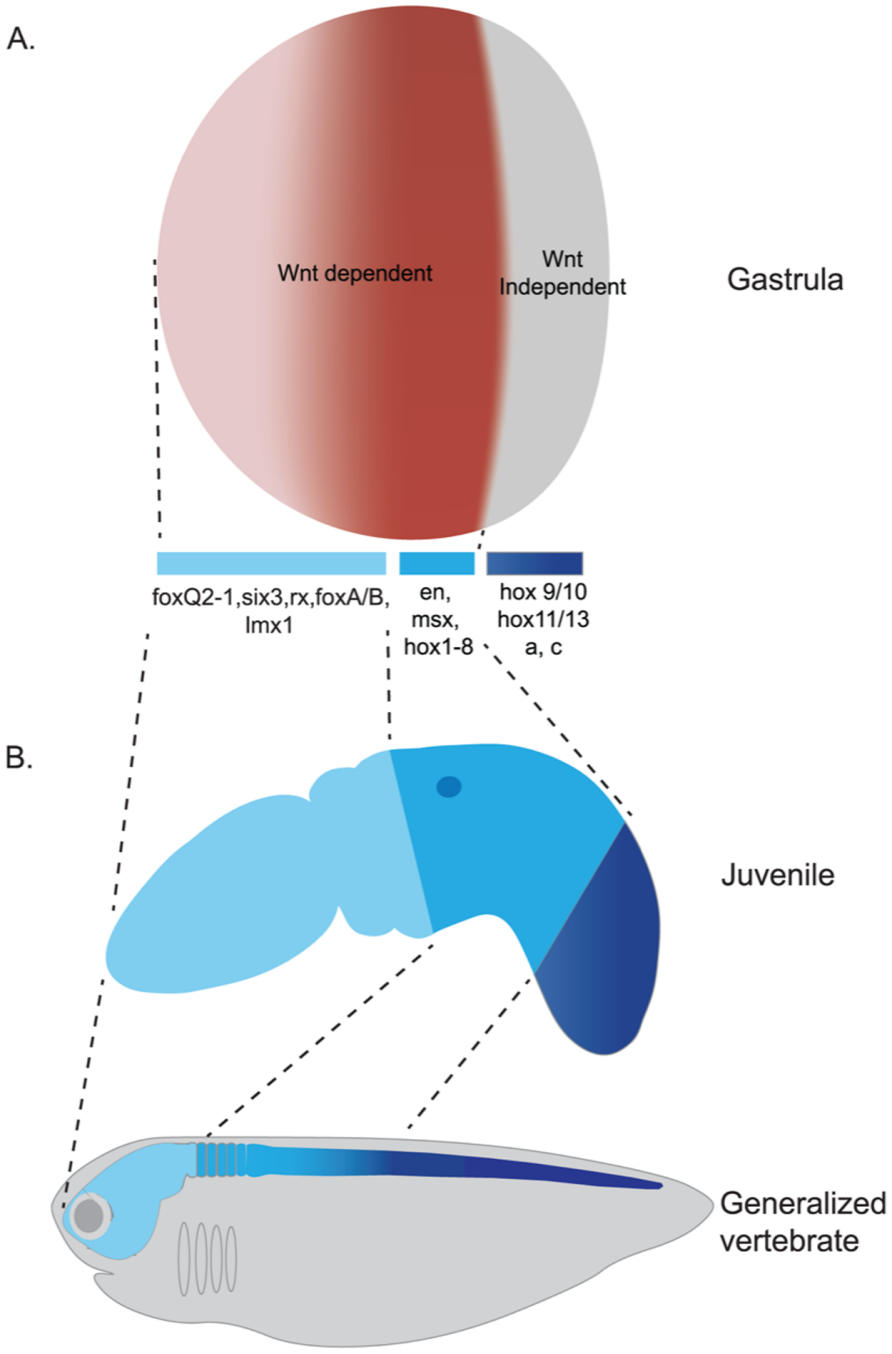
Summary model for the role of cWnt signaling in patterning the A/P axis of *S*. *kowalevskii*. (A), At the gastrula stage, the embryonic ectoderm is two main domains. The most posterior region around the blastopore is defined by posterior Hox, which is insensitive to cWnt signaling during the initial establishment of the A/P axis. The anterior boundary of the posterior ectoderm is at the anterior limit of *hox9/10* expression. (B), The transcriptional similarities in A/P patterning genes are demonstrated in this comparison between a generalized vertebrate and an early juvenile of *S*. *kowalevskii*, showing how the gene networks regulated by cWnt are very similar between the two taxa. A/P, anteroposterior; cWnt, canonical Wnt.

These targeted β-catenin siRNA experiments further support that there is a threshold of Wnt sensitivity in the trunk ectoderm. Wnt inhibition is sufficient to ectopically expand the most anterior genes, but only the presumptive anterior ectoderm can expand following cWnt suppression. Anterior/midtrunk gene expression requires active cWnt signaling, whereas posterior genes such as *hox9/10* are unaffected by suppression of cWnt during the early establishment of A/P pattern (Fig 11). The inability to anteriorize posterior ectoderm by elimination of Wnt signaling, and the early activation of posterior genes even in the absence of cWnt signaling is likely to be due to non-cWnt posteriorizing signals emitted by endomesoderm.

## Discussion

### A full ancestral complement of Wnt ligands, antagonists, and Fz receptors in hemichordates

A variety of analyses from genome and EST projects have established that the ancestral bilaterian complement of Wnts was likely 13 distinct families. Comparisons between bilaterian clades have revealed both Wnt gene diversification and loss during bilaterian body plan evolution. Within the deuterostomes, in chordates, the ancestral complement of *Wnt* gene families is 12; amphioxus has 13 ligands, and humans have 19, which can be classified into 12 distinct subfamilies [83]. The only subgroup missing from the vertebrates and amphioxus is *WntA*. In the tunicates, data from ascidians suggest that several Wnts were lost during the extensive genomic rearrangements in the tunicate lineage. Outside of chordates, 11 Wnts have been isolated from sea urchins with *wnt2* and *11* absent from the genome of *S*. *purpuratus* [62], and RNAseq data suggests this is conserved in another species of urchin [72]. From comprehensive arthropod sampling and recent work on onychophorans, along with limited lophotrochozoan sampling, the likely ancestral repertoire of protostome Wnt genes was 12, excluding *wnt3* [84,85], with several groups showing secondary losses of individual ligands. The most parsimonious reconstruction of the Wnt gene family complement in bilaterians is 13. Sequence data from *S*. *kowalevskii* conclusively supports this hypothesis, and provides the first data for a bilaterian possessing all of the 13 Wnt ligand families.

Comparative studies from placozoans, sponges, and ctenophores suggest that two Fzs were ancestral to metazoans [6,13,79,86]. Data from sea urchins [62], sea stars [87], chordates, hemichordates (our study), and annelids [85] support the conclusions from cnidarians that the Fz complement *fz5/8*, *fz1/2/7*, *fz4*, and *fz9/10* is the ancestral state for bilaterians and eumetazoans, and that the Fz3/6 group present in chordates represents a lineage-specific duplication from the Fz1/2/7 group [62,79].

We cloned and characterized a range of extracellular Wnt modifiers. Of the characterized Wnt antagonists, we cloned *dkk1/2/4*, *dkk3*, *sfrp1/5*, *sfrp3/4* (frzb), *WISE/sclerostin*, *wnt inhibitory factor* (wif), and *notum/wingful*, and of the Wnt agonists, we cloned *r-spondin*. Comparative genome surveys that explicitly discuss extracellular Wnt modifiers are scarce, and outside of chordates there are few reports of the presence or absence of these genes in other groups. Notably, a comprehensive survey in sea urchins [62] has identified all the Wnt modifiers that we identified except *sclerostin*, and thus *S*. *kowalevskii* has many of the antagonists that have been described in a variety of vertebrate species.

### An early role of cWnt signaling in the establishment of the A/P axis in hemichordates

The current data from hemichordates, when combined with data from other metazoans, provides strong support for the hypothesis that cWnt signaling was intimately involved in the early evolution of the A/P axis. In many bilaterian groups, Wnts have been proposed to play a conserved role in anterior suppression and posteriorization, and strong supporting experimental evidence has been generated from chordates, sea urchin, annelids, planarians, and acoels [20,23,34,37–40,43] (reviewed in [10,11,14]). Functional data from cnidarians also demonstrates that the evolution of the role of Wnts in axis polarization was likely a very deep innovation of metazoan embryos [77,88–90], even prior to bilaterians. We present comprehensive expression data for the entire complement of Wnt ligands throughout all early developmental stages (Figs 2 and 3), as well as of Wnt modifiers (antagonists and agonists) (Fig 4, S3 Fig) and Fzs (Fig 5). The relative expression domains of Wnt ligands and their antagonists resemble those of other bilaterians at opposite ends of the forming axis (Fig 6). In *S*. *kowalevskii*, the predominant expression of Wnt ligands posteriorly, excluded from the most anterior domains, the broad expression of the four *fz* genes in localized ectodermal domains down the A/P axis, and the predominant localization of Wnt antagonists in the anterior of the embryo, resembles expression patterns of many different metazoan embryos [11]. However, our data contrast with the comprehensive expression data from sea urchins, which shows most of the ligand expression domains in early development in the endomesoderm, with a subset of the ligands with localized domains in the ectoderm [72]. In this study, we focus on the role of Wnts in ectodermal patterning, but it is important to note that Wnt ligands, Fzs, and Wnt modifiers are also expressed in the endoderm and mesoderm (Figs 2, 3 and 5), and likely also play important developmental roles in those germ layers (S4 Fig). Although the relative localization of the ligands and their antagonists during A/P patterning is perhaps not surprising based on other comparative studies, the functional data we present, both embryological and molecular, show some unexpected results. While we have not addressed the function of individual Wnt ligands or identified which ligands activate the cWnt pathway, our data reveal close similarities to the regulatory role of cWnt during vertebrate neuraxis A/P patterning that are not shared by invertebrate chordates, and some surprising results for the role of cWnt in posterior specification.

In both vertebrates and *S*. *kowalevskii*, the ectoderm is initially fated to become anterior unless exposed to posteriorizing signals from the endomesoderm. Early embryonic manipulation of cWnt signaling, either positively or negatively, results in dramatic and complementary changes to the relative proportions of the major body regions, the proboscis, collar, and trunk (Figs 7 and 10). Over-activation of the pathway or overexpression of Wnt3 ligand results in reduction or loss of the proboscis and anterior collar (Fig 7), and overexpression of secreted antagonists of the cWnt pathway results in an over expansion of the anterior proboscis fates and reduction in the size of the trunk (Fig 10). The severity of anterior truncation from Wnt over-activation was concentration-dependent; a high level of 1-azakenpaullone resulted in loss of both anterior collar and proboscis fates, whereas lower concentrations resulted in loss of only the proboscis markers (Fig 7). This supports a model of a Wnt gradient, specifying the A/P pattern of the anterior embryo as has been proposed in vertebrates [20]. Despite the morphological disparity between vertebrates and hemichordates, cWnt is regulating the same region of the transcriptional network that defines the ectodermal A/P axis in both species. Proboscis/anterior collar markers, like forebrain/midbrain markers, are down-regulated, whereas posterior collar/pharynx/anterior trunk markers, like hindbrain markers, are up-regulated by cWnt signaling. The timing of Wnt activity during this early phase of A/P specification is also very similar between vertebrates and hemichordates. In *S*. *kowalevskii*, the onset of zygotic *Wnt* expression at midblastula (11 h) corresponds to the onset of posteriorizing activity. This was determined by timed treatments with the GSK3β inhibitor 1-azakenpaullone (S6 Fig). Treatments beginning at early blastula, just before the onset of zygotic Wnt expression, have the most severe anterior truncation, and this time period likely represents the onset of A/P patterning. Earlier treatments impact AV patterning and the amount of endomesoderm specified by β-catenin. Incremental delays of onset of exposure result in less severe anterior truncations through gastrulation, suggesting that the anterior ectoderm becomes increasingly refractive to cWnt with time.

### Defining identities along the A/P axis

A key finding of our work is the subdivision of the ectoderm into three domains regarding their sensitivity to cWnt (Fig 11). Below, we compare these observations with results from other metazoans.

### An ancient role in anterior repression

The *S*. *kowalevskii* anterior-most ectoderm is defined by a set of transcription factors (*six3*, *rx* and, *foxQ2-1*), whose anterior expression is conserved over a broad phylogenetic range [40]. Early cWnt activation or ligand overexpression leads to anterior marker inhibition and a “proboscis-less” embryo (Figs 7 and 8, S5 and S6 Figs), and overexpression of anteriorly localized Wnt antagonists results in expansion of this anterior territory (Fig 10, S7 Fig). This demonstrates that cWnt needs to be actively inhibited to allow correct patterning of anterior territories. However, the repressive effect of cWnt is likely tightly regulated in normal development in the anterior ectoderm to produce graded Wnt levels and correctly pattern proboscis and collar fates. qPCR data indicates that the most anterior fates are the most strongly inhibited by cWnt activity, with more caudal proboscis and collar markers less strongly down-regulated supportive of a local repressive gradient in the anterior ectoderm (Fig 8B). In addition, loss-of-function data suggest some anterior markers require some cWnt signaling for transcription; knock down of fz5/8 demonstrated that *rx* expression contracts posteriorly when Wnt signaling is locally repressed in the anterior ectoderm, and anterior clones of β-catenin-deficit cells fail to express *rx*, but activate ectopically the apical marker *FGFsk-1* [48]. Further, more sophisticated experiments would be required to further dissect this issue. Moreover, the initial broad expression of anterior markers in the ectoderm together with the observation that ectodermal cells separated from the endomesoderm at blastula stages develop into anterior-most ectoderm [15] suggests that anterior identity could correspond to a default or intrinsic fate. In comparison with vertebrates, it appears that the hemichordate embryo does not depend on an endomesodermal source of Wnt antagonists for anterior patterning; the anterior ectoderm suffices. The implication of cWnt in anterior suppression has been documented in a wide range of phylogenetically diverse groups of animals from chordates, echinoderms, arthropods, and annelids [20,36,40,41,97]. A similar situation may operate during regeneration in acoels and planarians and for the specification of the aboral pole in cnidarians [37–39,43,88].

### cWnt specifies midaxial identities

We have shown that cWnt is necessary and sufficient for the activation of markers of the midaxial identities in the posterior collar/anterior trunk along with anterior and central class Hox genes (Figs 8 and 9). This territory corresponds transcriptionally to the chordate hindbrain and anterior spinal cord [47]. Although anterior suppression has been broadly demonstrated in bilaterians, a role for cWnt in promotion of more posterior territories by up-regulation of midaxial markers, rather than by repression of anterior ones, comes mainly from vertebrates in which Wnts promote hindbrain and anterior spinal cord fates. We propose that this region of both embryos represents a homologous embryonic midaxial territory and is regulated by a cWnt-dependent regulatory program. Importantly, this conclusion could not have been strongly supported with current knowledge from nonvertebrate deuterosomes. In indirect-developing larval species of echinoids, asteroids, and hemichordates, most of the larval ectoderm is fated to be anterior [34,91,92], without an equivalent midaxial trunk territory. Consequently, the experimental focus in echinoderms has been mainly on the establishment and patterning of the anterior neural territory [34,35]. Nevertheless, in urchin larvae, Wnt5 activates a posterior ectodermal marker in the larval ectodermal [93], suggesting some role of Wnt, even in a body plan without a trunk. Strong similarities have not been found for tunicates, and it is possible that this patterning device has been greatly modified or changed its function. In cephalochordates, however, cWnt activation by Gsk3β inhibitor treatments leads to an expected anterior truncation, but the reported effect is rather moderate, affecting only the most anterior region of the neural tube, and it is thought that most of the axis is patterned independent of cWnt signaling [29]. Our data is thus very similar to vertebrate neural data except that patterning influences the entire ectoderm rather than a localized central nervous system. We thus propose that cWnt has been instrumental in the specification of the anterior axial and midaxial identities at least at the base of the deuterostomes, and that secondary modifications have occurred in echinoderms and invertebrate chordate lineages. Outside of deuterostomes, there is support for a role of cWnt in trunk identity during planarian regeneration [94,95], but most other experimental studies in arthropods deal with posterior growth rather than initial establishment of the A/P axis [18].

### cWnt does not specify posterior axial identity

We have previously reported that in *S*. *kowalevskii*, endomesoderm sends posteriorizing signals to the ectoderm in mid/late blastula stages that would otherwise adopt an anterior identity [15]. Gain- and loss-of-function experiments presented here demonstrate that initiation and early expression of posterior trunk markers such as *hox9/10*, *hox11/13a*, *c*, are insensitive to cWnt signaling. Moreover, targeted knock down of β-catenin by siRNA starting at the 2-, 4-, and 8-cell stages only expands the expression domains of anterior markers down to a region corresponding to the midtrunk, suggesting that the posterior trunk is resistant to anteriorization by cWnt inhibition (Fig 10ii, S7 Fig). These results suggest that posterior ectoderm identity is specified independent of cWnt and that other factors are involved in specification of posterior trunk. In vertebrates, Nodal, bone morphogenetic protein, fibroblast growth factor, and retinoic acid signaling are all involved in this process [96,97] and we are currently investigating the roles of these pathways during the early development of *S*. *kowalevskii*.

By combining the results of both gain- and loss-of-function, we tentatively mapped the boundary between cWnt-dependent and cWnt-independent ectodermal domains at the anterior limit of *hox9/10* expression (Fig 11). Based on similarities in gene expression, this limit roughly corresponds to the posterior spinal cord in vertebrates. Our results are very similar to what is observed in comparable experiments performed in vertebrates (reviewed in [27,98]) When *X*. *laevis* animal caps are cut following RNA injection of *xwnt3a* and *noggin*, the forebrain markers *XAG-1*, *xanf-2*, and *otxA* were inhibited, and the hindbrain markers markers *en-2* and *krox-20* were induced. However, this approach does not activate the spinal cord marker *hoxB9* [22,99]. Moreover, when the Wnt pathway is activated in whole *X*. *laevis* or chick embryos, it results in posteriorization of the anterior neural plate and expansion of hindbrain markers, whereas repression of cWnt has the reciprocal phenotype; repression of hindbrain fates and expansion of forebrain fates. However, posterior spinal cord markers were not examined in these studies, making it difficult to make firm conclusions about the role of Wnt signaling in posterior neural territories [20,22,23,25,96,99,100]. Our results suggest that the early specification of deuterostome posterior embryonic fates may be more dependent on the activity of other secreted ligands and that cWnt plays a less important role than previously proposed.

### Discrete conserved roles of Wnts during early axial patterning

We argue that the consideration of the role of cWnt signaling in posteriorization largely conflates several developmentally distinct roles of Wnts during AV and A/P patterning. Three distinct roles of the cWnt pathway in early embryonic axial patterning of metazoans have been proposed to have deep evolutionary origins in animal evolution (reviewed in [10,11,14]), and so far no single species displays all three of these. A clear developmental distinction may help for making coherent hypotheses about the role of this complex pathway in the establishment of metazoan axes.

The role with the earliest onset in development involves the action of β-catenin in the establishment of the AV axis and the specification of the endomesoderm. This role has been described in sea urchins, hemichordates, cnidarians, and nemerteans [15–17,101]. We described elsewhere this conserved function in *S*. *kowalevskii* [15]. Importantly, we have shown that endomesoderm specification by β-catenin is essential for subsequent ectoderm A/P patterning. We propose that endomesoderm sends posteriorizing signals to the overlying ectoderm that on its own harbors a “default” anterior fate that is independent of Wnt signaling. These unidentified signals are likely controlling posterior identity and posterior trunk formation, and are responsible for the initial cWnt insensitivity of this part of the embryo (Fig 11).

The second role is dealt with explicitly in this study and involves the role of cWnt in the early establishment of the A/P axis. The role of cWnt in anterior suppression has broad phylogenetic support. In deuterostomes, posteriorization of ectodermal fates by cWnt activity has been firmly established in vertebrate CNS in multiple species [20,23,97], and demonstrated to a limited extent in echinoids [93], and now in hemichordates. Whether this function of cWnt is broadly conserved in bilaterians is still unclear because it has not been formally investigated during embryogenesis in animals in which anterior suppression is evidenced; however, work in planarian and acoel regeneration are supportive of a broader role in posterior specification [37,38,43,94].

Finally, the third role of the cWnt pathway is later in development, following the early crude establishment of the A/P axis. Both arthropods and vertebrates deploy a conserved regulatory network of genes localized in a terminal growth zone mediated by the action of Wnts, which likely represents an ancestral developmental strategy for posterior growth (reviewed in [11,18]). We are currently investigating a later role of Wnts in posterior extension of the trunk in hemichordates. Following the early establishment of A/P polarity, hemichordates undergo an extended period of posterior growth to elongate the trunk. Wnt genes continue to be expressed in the posterior and potentially mediate this morphological extension, which mechanistically may be homologous to posterior growth in arthropods and chordates. If this is supported by further experiments, then early development in *S*. *kowalevskii* would be regulated by all three of the proposed conserved roles of cWnt signaling in axial patterning, endomesoderm specification, early establishment of A/P axis, and posterior growth.

## Supporting information

S1 Movie Overexpression of sfrp1/5 by mRNA injection. The embryo at three days of development displays an enlarged proboscis and reduced collar and trunk. sfrp, secreted frizzled-related protein.
(MOV)

S2 Movie Stage control for *sfrp1/5* overexpression. sfrp, secreted frizzled-related protein.
(MOV)

S1 Text Cloning and expression of Wnt modifiers: Agonists and antagonists. (DOCX)

S1 Data Raw data for qPCR Fig 8A. qPCR, quantitative PCR.
(XLSX)

S2 Data Raw data for qPCR Fig 8B. qPCR, quantitative PCR.
(XLSX)

S1 Fig Phylogenetic trees for Wnt, Fz, cysteine knot-containing and Sfrp proteins. Bf, *B*. *floridae*; Ci, *Ciona intestinalis*; Dr, *Danio rerio;* Fz, frizzled; Gg, *Gallus gallus*; Hs, *H*. *sapiens*; Mm, *M*. *musculus*; Nv, *Nematostella vectensis*; Sfrp, secreted frizzled-related protein; Sk, *S*. *kowalevskii*; Sp, *Strongylocentrotus purpuratus*; Xl, *X*. *laevis*; Xt, *X*. *tropicalis*.
(TIF)

S2 Fig Phylogenetic trees for Dkk, R-spondin, Notum/wingful, and Wif proteins. Bf, *B*. *floridae*; Bm, *Bombyx mori*; Ci, *C*. *intestinalis*; Cq, *Culex quiquefasciatus*; Dkk, Dickkopf; Dm, *Drosophila melanogaster*; Dr, *Danio rerio*; Gg, *G*. *gallus*; Hs, *H*. *sapiens*; Mm, *Mus musculus*; Nv, *N*. *vectensis*; Sk, *S*. *kowalevskii*; Sp, *S*. *purpuratus*; Tc, *Tribolium castaneum*; Wif, wnt inhibitory factor; Xl; X. laevis; Xt, *X*. *tropicalis*.
(TIF)

S3 Fig Expression of Wnt modifiers: Antagonists and agonists. Whole mount in situ hybridization of Wnt modifier genes. All data are presented as optical sagittal or frontal sections following clearing in Murray Clear. Developmental staging is from blastula to 72 h of development. All embryos are oriented with anterior, or animal (in the case of blastula), to the top left of the panel and posterior, or vegetal, to the bottom right of the panel. Right column, ventral is to the bottom left. Unless otherwise noted, expression is ectodermal. (A), Expression of *dkk3* at blastula (Ai), at late gastrula (Aii), at 48 h, surface view of an uncleared embryo (Aiii), at 60 h, side view (Aiv), and at 72 h of development in side view (Av). (B), Expression of *wif* at late gastrula (Bi), at 36 h (Bii), at 48 h (Biii-iv), and at 72 h of development (Bv). (C), Expression of *notum* at blastula (Ci), at late gastrula (Cii), at 48 h (Ciii), at 60 h, ventral view (Civ), and at 72 h of development (Cv). (D), Expression of *sclerostin* at blastula (Di), at early gastrula (Dii), at 36 h, white arrow indicates anterior endodermal expression (Diii), and at 60 h of development in dorsal view, with the focal plane through the dorsal ectoderm. Arrows indicate expression along the dorsal midline (Div), and at 72 h of development in side view (Dv). (E), Expression of *r-spondin* at blastula (Ei), at midgastrula (Eii), at late gastrula (Eiii), at 48 h in side view (Eiv), and at 60 h of development (Ev). *wif*, *wnt inhibitory factor*.
(TIF)

S4 Fig Activation of the cWnt pathway posteriorizes the endomesoderm. Embryos were treated with 10 μM of 1-azakenpaullone from midblastula stages (15.5 h) until fixation at 30 h of development. In situ hybridization for *foxA* (A and B) and *caudal* (C and D). Both markers show an anterior extension in their endomesodermal expression. DMSO-treated control embryos (A and C). 1-azakenpaullone-treated embryos (B and D). Anterior to the top. Arrows indicate the anterior limit of expression in the endomesoderm. cWnt, canonical Wnt; DMSO, dimethyl sulfoxide.
(TIF)

S5 Fig Activation of the Wnt pathway by recombinant murine Wnt3a protein posteriorizes the embryo. Embryos were treated with 200 ng/ml of Wnt3a protein from 1-cell stage to midgastrula stages, and fixed immediately (A-H) or at the end of gastrulation (I-N). Such a treatment led to a similar phenotype to what was observed upon *wnt3* mRNA injection or 1-azakenpaullone treatment. Early endomesoderm specification was not affected as revealed by the internal expression of *otx* (C, D) and *foxA* (G, H), whereas the endodermal expression of *foxA* expanded into presumptive proboscis mesoderm at later stages (white arrow in M and N). Posterior ectodermal expression of *hox9/10* was unchanged (E, F). Anterior ectodermal markers *six3* (A, B) and *foxQ2-1* (I, J) were repressed. Intermediate ectodermal markers expression was expanded and shifted anteriorly: ectodermal ring of *otx* (white arrows in C and D) and *engrailed* (K, L). Anterior is to the top.
(TIF)

S6 Fig Gradual anterior truncations according to the onset of cWnt pathway activation. (A), Experimental scheme: embryos were treated with 10 μM of 1-azakenpaullone starting the treatment every 2 h and ending at 64 h of development. Embryos were fixed at early gastrula stages and at 96 h. (B-D), At gastrula stages, no morphological defects are detected, but ectodermal A/P marker expression is modified. While the expression of the anterior-most marker *sfrp1/5* (B) is abolished, except for the latest treatment, in which a weaker expression is detected, the initial loss of the anterior marker *six3/6* (C) is progressively recovered when the treatment is delayed. The posterior marker *hox9/10* (D) expression is unchanged. (E-I), At four days of development, the morphology is dramatically affected; when embryos are treated early (11 h), they are truncated down to the anterior trunk. A full range of progressively less severe truncations are observed when the treatment is delayed until 19 h. Expression of the proboscis marker *six3* (E), the anterior trunk marker *en* (F), the trunk marker *msx* (G), and the posterior trunk marker *hox9/10* (H). (I), Schematic interpretation of the phenotypes; gray area corresponds to the truncated region. (i), DMSO-treated control embryos. 1-azakenpaullone treatment started at 11h (ii), 13 h (iii), 15 h (iv), 17h (v), and 19 h (vi). Gastrulae (B-D) are lateral view of hemi-sectioned embryos with animal to the top. Four days old embryos (E-H) are shown in lateral view with anterior to the top and ventral to the left, except inset in Fiv and Giv that show top views of the anterior of the embryo. A/P, anteroposterior; cWnt, canonical Wnt; DMSO, dimethyl sulfoxide.
(TIF)

S7 Fig Targeted β-catenin siRNA injection experiments. (A-F), Models of targeted injections, red indicating the targeted blastomere for injection. Data panels below each model represent embryos injected at 1-, 2-, 4-, or 8-cell stage. (G-J), Effect of injections on the endodermal marker *foxA*. (K-P), Effect of injection on the expression of the apical marker *foxQ2-1*. siRNA, short interfering RNA.
(TIF)

## Abbreviations

A/P: anteroposterior
AV: animal/vegetal
cWnt: canonical Wnt
D/V: dorsoventral
Dkk: Dickkopf
EST: expressed sequence tag
Fz: frizzled
GSK3β: glycogen synthase kinase 3 beta
hpf: h postfertilization
lef: lymphoid enhancer factor
qPCR: quantitative PCR
RT-PCR: reverse transcription PCR
Sfrp: secreted frizzled-related protein
siRNA: short interfering RNA
Tcf: T-cell factor
*wif*: *wnt inhibitory factor*

## References

[1] Logan CY, Nusse R. The Wnt signaling pathway in development and disease. Annu Rev Cell Dev Biol. 2004;20:781–810. doi: 10.1146/annurev.cellbio.20.010403.113126 . 15473860 10.1146/annurev.cellbio.20.010403.113126

[2] MacDonald BT, Tamai K, He X. Wnt/beta-catenin signaling: components, mechanisms, and diseases. Developmental cell. 2009;17(1):9–26. Epub 2009/07/22. doi: 10.1016/j.devcel.2009.06.016 . 19619488 10.1016/j.devcel.2009.06.016 PMC2861485

[3] Adamska M, Degnan SM, Green KM, Adamski M, Craigie A, Larroux C, et al Wnt and TGF-beta Expression in the Sponge Amphimedon queenslandica and the Origin of Metazoan Embryonic Patterning. PloS one. 2007;2(10):e1031 doi: 10.1371/journal.pone.0001031 . 17925879 10.1371/journal.pone.0001031 PMC2000352

[4] Adamska M, Larroux C, Adamski M, Green K, Lovas E, Koop D, et al Structure and expression of conserved Wnt pathway components in the demosponge Amphimedon queenslandica. Evolution & development. 2010;12(5):494–518. Epub 2010/10/05. doi: 10.1111/j.1525-142X.2010.00435.x . 20883218 10.1111/j.1525-142X.2010.00435.x

[5] Kusserow A, Pang K, Sturm C, Hrouda M, Lentfer J, Schmidt HA, et al Unexpected complexity of the Wnt gene family in a sea anemone. Nature. 2005;433(7022):156–60. doi: 10.1038/nature03158 . 15650739 10.1038/nature03158

[6] Srivastava M, Simakov O, Chapman J, Fahey B, Gauthier ME, Mitros T, et al The Amphimedon queenslandica genome and the evolution of animal complexity. Nature. 2010;466(7307):720–6. Epub 2010/08/06. doi: 10.1038/nature09201 . 20686567 10.1038/nature09201 PMC3130542

[7] Ryan JF, Baxevanis AD. Hox, Wnt, and the evolution of the primary body axis: insights from the early-divergent phyla. Biol Direct. 2007;2:37 Epub 2007/12/15. doi: 10.1186/1745-6150-2-37 . 18078518 10.1186/1745-6150-2-37 PMC2222619

[8] Guder C, Philipp I, Lengfeld T, Watanabe H, Hobmayer B, Holstein TW. The Wnt code: cnidarians signal the way. Oncogene. 2006;25(57):7450–60. doi: 10.1038/sj.onc.1210052 . 17143289 10.1038/sj.onc.1210052

[9] Martindale MQ, Hejnol A. A developmental perspective: changes in the position of the blastopore during bilaterian evolution. Developmental cell. 2009;17(2):162–74. Epub 2009/08/19. doi: 10.1016/j.devcel.2009.07.024 . 19686678 10.1016/j.devcel.2009.07.024

[10] Niehrs C. On growth and form: a Cartesian coordinate system of Wnt and BMP signaling specifies bilaterian body axes. Development. 2010;137(6):845–57. Epub 2010/02/25. doi: 10.1242/dev.039651 . 20179091 10.1242/dev.039651

[11] Petersen CP, Reddien PW. Wnt signaling and the polarity of the primary body axis. Cell. 2009;139(6):1056–68. Epub 2009/12/17. doi: 10.1016/j.cell.2009.11.035 . 20005801 10.1016/j.cell.2009.11.035

[12] Lapebie P, Gazave E, Ereskovsky A, Derelle R, Bezac C, Renard E, et al WNT/beta-Catenin Signalling and Epithelial Patterning in the Homoscleromorph Sponge Oscarella. PloS one. 2009;4(6):e5823 Epub 2009/06/09. doi: 10.1371/journal.pone.0005823 . 19503791 10.1371/journal.pone.0005823 PMC2688036

[13] Pang K, Ryan JF, Program NCS, Mullikin JC, Baxevanis AD, Martindale MQ. Genomic insights into Wnt signaling in an early diverging metazoan, the ctenophore Mnemiopsis leidyi. EvoDevo. 2010;1(1):10 doi: 10.1186/2041-9139-1-10 . 20920349 10.1186/2041-9139-1-10 PMC2959043

[14] Loh KM, van Amerongen R, Nusse R. Generating Cellular Diversity and Spatial Form: Wnt Signaling and the Evolution of Multicellular Animals. Developmental cell. 2016;38(6):643–55. doi: 10.1016/j.devcel.2016.08.011 . 27676437 10.1016/j.devcel.2016.08.011

[15] Darras S, Gerhart J, Terasaki M, Kirschner M, Lowe CJ. beta-catenin specifies the endomesoderm and defines the posterior organizer of the hemichordate Saccoglossus kowalevskii. Development. 2011;138(5):959–70. doi: 10.1242/dev.059493 . 21303849 10.1242/dev.059493 PMC3035098

[16] Henry JQ, Perry KJ, Wever J, Seaver E, Martindale MQ. Beta-catenin is required for the establishment of vegetal embryonic fates in the nemertean, Cerebratulus lacteus. Developmental biology. 2008;317(1):368–79. Epub 2008/04/05. doi: 10.1016/j.ydbio.2008.02.042 . 18387602 10.1016/j.ydbio.2008.02.042

[17] Wikramanayake AH, Huang L, Klein WH. beta-Catenin is essential for patterning the maternally specified animal-vegetal axis in the sea urchin embryo. Proceedings of the National Academy of Sciences of the United States of America. 1998;95(16):9343–8. Epub 1998/08/05. . 9689082 10.1073/pnas.95.16.9343 PMC21340

[18] Martin BL, Kimelman D. Wnt signaling and the evolution of embryonic posterior development. Current biology: CB. 2009;19(5):R215–9. Epub 2009/03/13. doi: 10.1016/j.cub.2009.01.052 . 19278640 10.1016/j.cub.2009.01.052 PMC5560127

[19] Christian JL, Moon RT. Interactions between Xwnt-8 and Spemann organizer signaling pathways generate dorsoventral pattern in the embryonic mesoderm of Xenopus. Genes Dev. 1993;7(1):13–28. Epub 1993/01/01. . 8422982 10.1101/gad.7.1.13

[20] Kiecker C, Niehrs C. A morphogen gradient of Wnt/beta-catenin signalling regulates anteroposterior neural patterning in Xenopus. Development. 2001;128(21):4189–201. . 11684656 10.1242/dev.128.21.4189

[21] Leyns L, Bouwmeester T, Kim SH, Piccolo S, De Robertis EM. Frzb-1 is a secreted antagonist of Wnt signaling expressed in the Spemann organizer. Cell. 1997;88(6):747–56. Epub 1997/03/21. . 9118218 10.1016/s0092-8674(00)81921-2 PMC3061830

[22] McGrew LL, Hoppler S, Moon RT. Wnt and FGF pathways cooperatively pattern anteroposterior neural ectoderm in Xenopus. Mech Dev. 1997;69(1–2):105–14. Epub 1998/03/05. . 9486534 10.1016/s0925-4773(97)00160-3

[23] Nordstrom U, Jessell TM, Edlund T. Progressive induction of caudal neural character by graded Wnt signaling. Nat Neurosci. 2002;5(6):525–32. Epub 2002/05/15. doi: 10.1038/nn854 . 12006981 10.1038/nn0602-854

[24] Piccolo S, Agius E, Leyns L, Bhattacharyya S, Grunz H, Bouwmeester T, et al The head inducer Cerberus is a multifunctional antagonist of Nodal, BMP and Wnt signals. Nature. 1999;397(6721):707–10. Epub 1999/03/06. doi: 10.1038/17820 . 10067895 10.1038/17820 PMC2323273

[25] Glinka A, Wu W, Delius H, Monaghan AP, Blumenstock C, Niehrs C. Dickkopf-1 is a member of a new family of secreted proteins and functions in head induction. Nature. 1998;391(6665):357–62. Epub 1998/02/05. doi: 10.1038/34848 . 9450748 10.1038/34848

[26] Houart C, Caneparo L, Heisenberg C, Barth K, Take-Uchi M, Wilson S. Establishment of the telencephalon during gastrulation by local antagonism of Wnt signaling. Neuron. 2002;35(2):255–65. Epub 2002/08/06. . 12160744 10.1016/s0896-6273(02)00751-1

[27] Elkouby YM, Frank D. Wnt/beta-Catenin Signaling in Vertebrate Posterior Neural Development Developmental Biology. San Rafael (CA) 2010. 21452442

[28] Yu JK, Satou Y, Holland ND, Shin IT, Kohara Y, Satoh N, et al Axial patterning in cephalochordates and the evolution of the organizer. Nature. 2007;445(7128):613–7. doi: 10.1038/nature05472 . 17237766 10.1038/nature05472

[29] Onai T, Lin HC, Schubert M, Koop D, Osborne PW, Alvarez S, et al Retinoic acid and Wnt/beta-catenin have complementary roles in anterior/posterior patterning embryos of the basal chordate amphioxus. Developmental biology. 2009;332(2):223–33. doi: 10.1016/j.ydbio.2009.05.571 . 19497318 10.1016/j.ydbio.2009.05.571

[30] Onai T, Takai A, Setiamarga DH, Holland LZ. Essential role of Dkk3 for head formation by inhibiting Wnt/beta-catenin and Nodal/Vg1 signaling pathways in the basal chordate amphioxus. Evolution & development. 2012;14(4):338–50. doi: 10.1111/j.1525-142X.2012.00552.x . 22765205 10.1111/j.1525-142X.2012.00552.x

[31] Auger H, Lamy C, Haeussler M, Khoueiry P, Lemaire P, Joly JS. Similar regulatory logic in Ciona intestinalis for two Wnt pathway modulators, ROR and SFRP-1/5. Developmental biology. 2009;329(2):364–73. doi: 10.1016/j.ydbio.2009.02.018 . 19248777 10.1016/j.ydbio.2009.02.018

[32] Imai KS, Hino K, Yagi K, Satoh N, Satou Y. Gene expression profiles of transcription factors and signaling molecules in the ascidian embryo: towards a comprehensive understanding of gene networks. Development. 2004;131(16):4047–58. doi: 10.1242/dev.01270 . 15269171 10.1242/dev.01270

[33] Lamy C, Rothbacher U, Caillol D, Lemaire P. Ci-FoxA-a is the earliest zygotic determinant of the ascidian anterior ectoderm and directly activates Ci-sFRP1/5. Development. 2006;133(15):2835–44. doi: 10.1242/dev.02448 . 16835437 10.1242/dev.02448

[34] Range RC, Angerer RC, Angerer LM. Integration of canonical and noncanonical Wnt signaling pathways patterns the neuroectoderm along the anterior-posterior axis of sea urchin embryos. PLoS Biol. 2013;11(1):e1001467 doi: 10.1371/journal.pbio.1001467 . 23335859 10.1371/journal.pbio.1001467 PMC3545869

[35] Range RC, Wei Z. An anterior signaling center patterns and sizes the anterior neuroectoderm of the sea urchin embryo. Development. 2016 doi: 10.1242/dev.128165 . 26952978 10.1242/dev.128165 PMC4909856

[36] Range RC, Wei Z. An anterior signaling center patterns and sizes the anterior neuroectoderm of the sea urchin embryo. Development. 2016;143(9):1523–33. doi: 10.1242/dev.128165 . 26952978 10.1242/dev.128165 PMC4909856

[37] Gurley KA, Rink JC, Sanchez Alvarado A. Beta-catenin defines head versus tail identity during planarian regeneration and homeostasis. Science. 2008;319(5861):323–7. Epub 2007/12/08. doi: 10.1126/science.1150029 . 18063757 10.1126/science.1150029 PMC2755502

[38] Petersen CP, Reddien PW. Smed-betacatenin-1 is required for anteroposterior blastema polarity in planarian regeneration. Science. 2008;319(5861):327–30. Epub 2007/12/08. doi: 10.1126/science.1149943 . 18063755 10.1126/science.1149943

[39] Iglesias M, Gomez-Skarmeta JL, Salo E, Adell T. Silencing of Smed-betacatenin1 generates radial-like hypercephalized planarians. Development. 2008;135(7):1215–21. Epub 2008/02/22. doi: 10.1242/dev.020289 . 18287199 10.1242/dev.020289

[40] Marlow H, Tosches MA, Tomer R, Steinmetz PR, Lauri A, Larsson T, et al Larval body patterning and apical organs are conserved in animal evolution. BMC biology. 2014;12:7 doi: 10.1186/1741-7007-12-7 . 24476105 10.1186/1741-7007-12-7 PMC3939940

[41] Fu J, Posnien N, Bolognesi R, Fischer TD, Rayl P, Oberhofer G, et al Asymmetrically expressed axin required for anterior development in Tribolium. Proceedings of the National Academy of Sciences of the United States of America. 2012;109(20):7782–6. doi: 10.1073/pnas.1116641109 . 22552230 10.1073/pnas.1116641109 PMC3356623

[42] Harterink M, Kim DH, Middelkoop TC, Doan TD, van Oudenaarden A, Korswagen HC. Neuroblast migration along the anteroposterior axis of C. elegans is controlled by opposing gradients of Wnts and a secreted Frizzled-related protein. Development. 2011;138(14):2915–24. doi: 10.1242/dev.064733 . 21653614 10.1242/dev.064733 PMC3119304

[43] Srivastava M, Mazza-Curll KL, van Wolfswinkel JC, Reddien PW. Whole-Body Acoel Regeneration Is Controlled by Wnt and Bmp-Admp Signaling. Current biology: CB. 2014 doi: 10.1016/j.cub.2014.03.042 . 24768051 10.1016/j.cub.2014.03.042

[44] Cameron CB, Garey JR, Swalla BJ. Evolution of the chordate body plan: new insights from phylogenetic analyses of deuterostome phyla. Proceedings of the National Academy of Sciences of the United States of America. 2000;97(9):4469–74. . 10781046 10.1073/pnas.97.9.4469 PMC18258

[45] Bourlat SJ, Juliusdottir T, Lowe CJ, Freeman R, Aronowicz J, Kirschner M, et al Deuterostome phylogeny reveals monophyletic chordates and the new phylum Xenoturbellida. Nature. 2006;444(7115):85–8. doi: 10.1038/nature05241 . 17051155 10.1038/nature05241

[46] Wada H, Satoh N. Details of the evolutionary history from invertebrates to vertebrates, as deduced from the sequences of 18S rDNA. Proceedings of the National Academy of Sciences of the United States of America. 1994;91(5):1801–4. . 8127885 10.1073/pnas.91.5.1801 PMC43251

[47] Lowe CJ, Wu M, Salic A, Evans L, Lander E, Stange-Thomann N, et al Anteroposterior patterning in hemichordates and the origins of the chordate nervous system. Cell. 2003;113(7):853–65. . 12837244 10.1016/s0092-8674(03)00469-0

[48] Pani AM, Mullarkey EE, Aronowicz J, Assimacopoulos S, Grove EA, Lowe CJ. Ancient deuterostome origins of vertebrate brain signalling centres. Nature. 2012;483(7389):289–94. Epub 2012/03/17. doi: 10.1038/nature10838 . 22422262 10.1038/nature10838 PMC3719855

[49] Lowe CJ, Tagawa K, Humphreys T, Kirschner M, Gerhart J. Hemichordate embryos: procurement, culture, and basic methods. Methods Cell Biol. 2004;74:171–94. Epub 2004/12/04. . 15575607 10.1016/s0091-679x(04)74008-x

[50] Bateson W. The early stages in the development of Balanoglossus (sp. incert.). Quart J Micr Sci. 1884;24:208–36.

[51] Bateson W. The later stages in the development of Balanoglossus kowalevskii, with a suggestion as to the affinities of the Enteropneusta. Quart J Micr Sci. 1885;25:21–122.

[52] Colwin A, Colwin L. The normal embryology of *Saccoglossus kowalevskii*. J Morphol. 1953;92:401–53.

[53] Colwin AL, Colwin LH. Relationships between the egg and larva of saccoglossus kowalevskii (enteropneusta): Axes and planes; general prospective significance of the early blastomeres. Journal of Experimental Zoology. 1951;117(1):111–37.

[54] Kunick C, Lauenroth K, Leost M, Meijer L, Lemcke T. 1-Azakenpaullone is a selective inhibitor of glycogen synthase kinase-3 beta. Bioorg Med Chem Lett. 2004;14(2):413–6. Epub 2003/12/31. . 14698171 10.1016/j.bmcl.2003.10.062

[55] Lowe CJ, Terasaki M, Wu M, Freeman RM Jr, Runft L, Kwan K, et al Dorsoventral patterning in hemichordates: insights into early chordate evolution. PLoS Biol. 2006;4(9):e291 Epub 2006/08/29. doi: 10.1371/journal.pbio.0040291 . 16933975 10.1371/journal.pbio.0040291 PMC1551926

[56] Freeman RM Jr, Wu M, Cordonnier-Pratt MM, Pratt LH, Gruber CE, Smith M, et al cDNA sequences for transcription factors and signaling proteins of the hemichordate Saccoglossus kowalevskii: efficacy of the expressed sequence tag (EST) approach for evolutionary and developmental studies of a new organism. Biol Bull. 2008;214(3):284–302. doi: 10.2307/25470670 . 18574105 10.2307/25470670

[57] Thompson JD, Gibson TJ, Plewniak F, Jeanmougin F, Higgins DG. The CLUSTAL_X windows interface: flexible strategies for multiple sequence alignment aided by quality analysis tools. Nucleic Acids Res. 1997;25(24):4876–82. Epub 1998/02/28. . 9396791 10.1093/nar/25.24.4876 PMC147148

[58] Ronquist F, Huelsenbeck JP. MrBayes 3: Bayesian phylogenetic inference under mixed models. Bioinformatics. 2003;19(12):1572–4. Epub 2003/08/13. . 12912839 10.1093/bioinformatics/btg180

[59] L SD. PAUP*: phylogenetic analysis using parsimony (* and other methods). Version 4 ed Sunderland, MA: Sinauer Associates; 1999.

[60] Schmittgen TD, Livak KJ. Analyzing real-time PCR data by the comparative C(T) method. Nat Protoc. 2008;3(6):1101–8. . 18546601 10.1038/nprot.2008.73

[61] van Ooyen A, Kwee V, Nusse R. The nucleotide sequence of the human int-1 mammary oncogene; evolutionary conservation of coding and non-coding sequences. EMBO J. 1985;4(11):2905–9. Epub 1985/11/01. . 2998762 10.1002/j.1460-2075.1985.tb04021.x PMC554596

[62] Croce JC, Wu SY, Byrum C, Xu R, Duloquin L, Wikramanayake AH, et al A genome-wide survey of the evolutionarily conserved Wnt pathways in the sea urchin Strongylocentrotus purpuratus. Developmental biology. 2006;300(1):121–31. . 17069790 10.1016/j.ydbio.2006.08.04 PMC1780136

[63] Kawano Y, Kypta R. Secreted antagonists of the Wnt signalling pathway. J Cell Sci. 2003;116(Pt 13):2627–34. Epub 2003/05/31. doi: 10.1242/jcs.00623 . 12775774 10.1242/jcs.00623

[64] Adell T, Thakur AN, Muller WE. Isolation and characterization of Wnt pathway-related genes from Porifera. Cell Biol Int. 2007;31(9):939–49. Epub 2007/05/02. doi: 10.1016/j.cellbi.2007.03.003 . 17470402 10.1016/j.cellbi.2007.03.003

[65] Bastin BR, Chou HC, Pruitt MM, Schneider SQ. Structure, phylogeny, and expression of the frizzled-related gene family in the lophotrochozoan annelid Platynereis dumerilii. EvoDevo. 2015;6:37 doi: 10.1186/s13227-015-0032-4 . 26640641 10.1186/s13227-015-0032-4 PMC4669655

[66] Leimeister C, Bach A, Gessler M. Developmental expression patterns of mouse sFRP genes encoding members of the secreted frizzled related protein family. Mech Dev. 1998;75(1–2):29–42. Epub 1998/09/18. . 9739103 10.1016/s0925-4773(98)00072-0

[67] Tendeng C, Houart C. Cloning and embryonic expression of five distinct sfrp genes in the zebrafish Danio rerio. Gene expression patterns: GEP. 2006;6(8):761–71. Epub 2006/03/01. doi: 10.1016/j.modgep.2006.01.006 . 16504595 10.1016/j.modgep.2006.01.006

[68] Lopez-Rios J, Esteve P, Ruiz JM, Bovolenta P. The Netrin-related domain of Sfrp1 interacts with Wnt ligands and antagonizes their activity in the anterior neural plate. Neural Dev. 2008;3:19 Epub 2008/08/22. doi: 10.1186/1749-8104-3-19 . 18715500 10.1186/1749-8104-3-19 PMC2542364

[69] Duprez D, Leyns L, Bonnin MA, Lapointe F, Etchevers H, De Robertis EM, et al Expression of Frzb-1 during chick development. Mech Dev. 1999;89(1–2):179–83. Epub 1999/11/24. . 10559495 10.1016/s0925-4773(99)00206-3

[70] Hoang BH, Thomas JT, Abdul-Karim FW, Correia KM, Conlon RA, Luyten FP, et al Expression pattern of two Frizzled-related genes, Frzb-1 and Sfrp-1, during mouse embryogenesis suggests a role for modulating action of Wnt family members. Developmental dynamics: an official publication of the American Association of Anatomists. 1998;212(3):364–72. Epub 1998/07/22. doi: 10.1002/(SICI)1097-0177(199807)212:3<364::AID-AJA4>3.0.CO;2-F . 9671940 10.1002/(SICI)1097-0177(199807)212:3<364::AID-AJA4>3.0.CO;2-F

[71] Wang S, Krinks M, Moos M Jr. Frzb-1, an antagonist of Wnt-1 and Wnt-8, does not block signaling by Wnts -3A, -5A, or -11. Biochem Biophys Res Commun. 1997;236(2):502–4. doi: 10.1006/bbrc.1997.6995 . 9240469 10.1006/bbrc.1997.6995

[72] Robert N, Lhomond G, Schubert M, Croce JC. A comprehensive survey of wnt and frizzled expression in the sea urchin Paracentrotus lividus. Genesis. 2014;52(3):235–50. doi: 10.1002/dvg.22754 . 24550167 10.1002/dvg.22754

[73] Rottinger E, DuBuc TQ, Amiel AR, Martindale MQ. Nodal signaling is required for mesodermal and ventral but not for dorsal fates in the indirect developing hemichordate, Ptychodera flava. Biology open. 2015;4(7):830–42. doi: 10.1242/bio.011809 . 25979707 10.1242/bio.011809 PMC4571091

[74] Mao B, Niehrs C. Kremen2 modulates Dickkopf2 activity during Wnt/LRP6 signaling. Gene. 2003;302(1–2):179–83. . 12527209 10.1016/s0378-1119(02)01106-x

[75] Mao B, Wu W, Li Y, Hoppe D, Stannek P, Glinka A, et al LDL-receptor-related protein 6 is a receptor for Dickkopf proteins. Nature. 2001;411(6835):321–5. Epub 2001/05/18. doi: 10.1038/35077108 . 11357136 10.1038/35077108

[76] Lee PN, Pang K, Matus DQ, Martindale MQ. A WNT of things to come: evolution of Wnt signaling and polarity in cnidarians. Semin Cell Dev Biol. 2006;17(2):157–67. Epub 2006/06/13. doi: 10.1016/j.semcdb.2006.05.002 . 16765608 10.1016/j.semcdb.2006.05.002

[77] Guder C, Pinho S, Nacak TG, Schmidt HA, Hobmayer B, Niehrs C, et al An ancient Wnt-Dickkopf antagonism in Hydra. Development. 2006;133(5):901–11. doi: 10.1242/dev.02265 . 16452091 10.1242/dev.02265

[78] Huang HC, Klein PS. The Frizzled family: receptors for multiple signal transduction pathways. Genome biology. 2004;5(7):234 doi: 10.1186/gb-2004-5-7-234 . 15239825 10.1186/gb-2004-5-7-234 PMC463283

[79] Schenkelaars Q, Fierro-Constain L, Renard E, Hill AL, Borchiellini C. Insights into Frizzled evolution and new perspectives. Evolution & development. 2015;17(2):160–9. doi: 10.1111/ede.12115 . 25801223 10.1111/ede.12115

[80] Quinlan R, Graf M, Mason I, Lumsden A, Kiecker C. Complex and dynamic patterns of Wnt pathway gene expression in the developing chick forebrain. Neural Dev. 2009;4:35 Epub 2009/09/08. doi: 10.1186/1749-8104-4-35 . 19732418 10.1186/1749-8104-4-35 PMC2757023

[81] Kikuchi A, Yamamoto H, Kishida S. Multiplicity of the interactions of Wnt proteins and their receptors. Cell Signal. 2007;19(4):659–71. Epub 2006/12/26. doi: 10.1016/j.cellsig.2006.11.001 . 17188462 10.1016/j.cellsig.2006.11.001

[82] van Amerongen R, Nusse R. Towards an integrated view of Wnt signaling in development. Development. 2009;136(19):3205–14. Epub 2009/09/09. doi: 10.1242/dev.033910 . 19736321 10.1242/dev.033910

[83] Garriock RJ, Warkman AS, Meadows SM, D’Agostino S, Krieg PA. Census of vertebrate Wnt genes: isolation and developmental expression of Xenopus Wnt2, Wnt3, Wnt9a, Wnt9b, Wnt10a, and Wnt16. Developmental dynamics: an official publication of the American Association of Anatomists. 2007;236(5):1249–58. doi: 10.1002/dvdy.21156 . 17436276 10.1002/dvdy.21156

[84] Hogvall M, Schonauer A, Budd GE, McGregor AP, Posnien N, Janssen R. Analysis of the Wnt gene repertoire in an onychophoran provides new insights into the evolution of segmentation. EvoDevo. 2014;5(1):14 doi: 10.1186/2041-9139-5-14 . 24708787 10.1186/2041-9139-5-14 PMC4021614

[85] Cho SJ, Valles Y, Giani VC Jr, Seaver EC, Weisblat DA. Evolutionary dynamics of the wnt gene family: a lophotrochozoan perspective. Mol Biol Evol. 2010;27(7):1645–58. Epub 2010/02/24. doi: 10.1093/molbev/msq052 . 20176615 10.1093/molbev/msq052 PMC2912473

[86] Srivastava M, Begovic E, Chapman J, Putnam NH, Hellsten U, Kawashima T, et al The Trichoplax genome and the nature of placozoans. Nature. 2008;454(7207):955–60. doi: 10.1038/nature07191 . 18719581 10.1038/nature07191

[87] McCauley BS, Akyar E, Filliger L, Hinman VF. Expression of wnt and frizzled genes during early sea star development. Gene expression patterns: GEP. 2013;13(8):437–44. doi: 10.1016/j.gep.2013.07.007 . 23899422 10.1016/j.gep.2013.07.007

[88] Marlow H, Matus DQ, Martindale MQ. Ectopic activation of the canonical wnt signaling pathway affects ectodermal patterning along the primary axis during larval development in the anthozoan Nematostella vectensis. Developmental biology. 2013;380(2):324–34. doi: 10.1016/j.ydbio.2013.05.022 . 23722001 10.1016/j.ydbio.2013.05.022 PMC4792810

[89] Momose T, Derelle R, Houliston E. A maternally localised Wnt ligand required for axial patterning in the cnidarian Clytia hemisphaerica. Development. 2008;135(12):2105–13. doi: 10.1242/dev.021543 . 18480163 10.1242/dev.021543

[90] Momose T, Houliston E. Two oppositely localised frizzled RNAs as axis determinants in a cnidarian embryo. PLoS Biol. 2007;5(4):e70 doi: 10.1371/journal.pbio.0050070 . 17355179 10.1371/journal.pbio.0050070 PMC1820609

[91] Gonzalez P, Uhlinger KR, Lowe CJ. The Adult Body Plan of Indirect Developing Hemichordates Develops by Adding a Hox-Patterned Trunk to an Anterior Larval Territory. Current biology: CB. 2017;27(1):87–95. doi: 10.1016/j.cub.2016.10.047 . 27939313 10.1016/j.cub.2016.10.047

[92] Yankura KA, Martik ML, Jennings CK, Hinman VF. Uncoupling of complex regulatory patterning during evolution of larval development in echinoderms. BMC biology. 2010;8:143 doi: 10.1186/1741-7007-8-143 . 21118544 10.1186/1741-7007-8-143 PMC3002323

[93] McIntyre DC, Seay NW, Croce JC, McClay DR. Short-range Wnt5 signaling initiates specification of sea urchin posterior ectoderm. Development. 2013;140(24):4881–9. doi: 10.1242/dev.095844 . 24227654 10.1242/dev.095844 PMC3848187

[94] Lander R, Petersen CP. Wnt, Ptk7, and FGFRL expression gradients control trunk positional identity in planarian regeneration. Elife. 2016;5 doi: 10.7554/eLife.12850 . 27074666 10.7554/eLife.12850 PMC4865369

[95] Scimone ML, Cote LE, Rogers T, Reddien PW. Two FGFRL-Wnt circuits organize the planarian anteroposterior axis. Elife. 2016;5 doi: 10.7554/eLife.12845 . 27063937 10.7554/eLife.12845 PMC4865367

[96] Kudoh T, Wilson SW, Dawid IB. Distinct roles for Fgf, Wnt and retinoic acid in posteriorizing the neural ectoderm. Development. 2002;129(18):4335–46. . 12183385 10.1242/dev.129.18.4335

[97] Schier AF, Talbot WS. Molecular genetics of axis formation in zebrafish. Annu Rev Genet. 2005;39:561–613. doi: 10.1146/annurev.genet.37.110801.143752 . 16285872 10.1146/annurev.genet.37.110801.143752

[98] Carron C, Shi DL. Specification of anteroposterior axis by combinatorial signaling during Xenopus development. Wiley Interdiscip Rev Dev Biol. 2016;5(2):150–68. doi: 10.1002/wdev.217 . 26544673 10.1002/wdev.217

[99] McGrew LL, Lai CJ, Moon RT. Specification of the anteroposterior neural axis through synergistic interaction of the Wnt signaling cascade with noggin and follistatin. Developmental biology. 1995;172(1):337–42. Epub 1995/11/01. doi: 10.1006/dbio.1995.0027 . 7589812 10.1006/dbio.1995.0027

[100] Kim CH, Oda T, Itoh M, Jiang D, Artinger KB, Chandrasekharappa SC, et al Repressor activity of Headless/Tcf3 is essential for vertebrate head formation. Nature. 2000;407(6806):913–6. Epub 2000/11/01. doi: 10.1038/35038097 . 11057671 10.1038/35038097 PMC4018833

[101] Wikramanayake AH, Hong M, Lee PN, Pang K, Byrum CA, Bince JM, et al An ancient role for nuclear beta-catenin in the evolution of axial polarity and germ layer segregation. Nature. 2003;426(6965):446–50. doi: 10.1038/nature02113 . 14647383 10.1038/nature02113

