# Supplementary material for: Anteroposterior axis patterning by early canonical Wnt signaling during hemichordate development
Source: PLoS Biol. 2018 Jan 16;16(1):e2003698. doi: 10.1371/journal.pbio.2003698 (PMC5786327; doi:10.1371/journal.pbio.2003698)
Supplement: S1 Text — (DOCX) [file pbio.2003698.s003.docx]

**Cloning and expression of Wnt modifiers: agonists and antagonists.**

**Dkk3**: The Dickkopf family of Wnt modulators were initially characterized in vertebrates and have been identified in echinoderms and cnidarians, suggesting an ancient metazoan origin [1,2]. However, they are absent in ecdysozoan genomes [3], and initial genomic screens of a limited number of lophotrochozoan species suggest that they may also be absent in this clade (personal communication David Weisblat UC Berkeley) suggesting secondary loss in the protostomes. The *dkk* family is deeply split into *dkk3* and *dkk1/2/4*. Sea urchins are known to have a copy of each gene, and we have cloned just one copy of each, a result supporting the conclusions of previous comparative studies (Fig. S2).

Although Dkk1/2/4 class of genes has a well established role in canonical Wnt antagonism [4], *dkk3* is not well characterized in any system, and there is no strong experimental evidence linking *dkk3* to Wnt antagonism. Some studies have linked it to Wnt agonism and to modulation of TGF-β signaling [5,6,7], but more work is needed to elucidate its role in animal development. Expression of the orthologue of *dkk3* in *S. kowalevskii* begins at the onset of gastrulation in isolated foci of cells throughout the ectoderm (Fig. S3A). The highest density of expressing cells is around the blastopore. As the embryo elongates following gastrulation, expression remains expressed in broadly dispersed ectodermal cells, but two regions are particularly densely clustered with expressing cells; at the apical tip of the proboscis and at the collar and anterior trunk (Fig. S3Aiii). This general distribution of expressing cells remains consistent throughout the later developmental stages we examined (Fig. S3Aiv-v).

**Wnt inhibitory factor.**

Wif, similar to Sfrp, is able to directly antagonize Wnt function by binding to Wnt ligands [8], though not the receptor. Again, there is little comparative data available for *wif*, but the data supports a conserved function during vertebrate evolution, with expression domains predominantly in the presomitic, unsegmented mesoderm and a lower level in the central nervous system. Expression is not detectable during gastrulation and begins only during somitogenesis in *Xenopus*, zebrafish and mouse. Over expression analysis demonstrates that its biological function is similar to FrzB, and results in hyper-anteriorized and dorsalized embryos [8]. The *wif* orthologue from *S. kowalevskii* exhibits quite a similar onset of expression; no transcripts are detected by whole mount *in situ* hybridization during gastrulation (Fig. S3Bi), and expression begins after mesoderm forms by enterocoely at 36 hrs. Expression by 48 hrs is detectable in all three mesodermal compartments (proboscis, collar, and trunk coeloms) (Fig. S3Biii, iv). By early juvenile stages, *wif* is expressed strongly in both the proboscis mesoderm and collar mesoderm, but is now downregulated in the trunk coelom. Expression is also detected in the forming 1st gill slit (Fig. S3Bv). Thus, in comparison to the other Wnt antagonists, *wif* is expressed in anterior locations but relatively late.

**Notum/wingful**: Notum/wingful is a secreted protein containing an esterase hydrolytic domain that regulates wingless/wnt1 during development by the release of glypicans and other GPI-anchored proteins from the cell surface [9,10,11]. It has been most comprehensively characterized in *Drosophila* wing disc development, and so far little comparative or functional characterization has been carried out in chordates except in cell culture [12,13]. During early development of *S. kowalevskii*, expression closely tracks the expression of *wnt1* (Fig. S3Ci). In *Drosophila*, *wingful* is positively regulated by the *wnt1* orthologue, *wingless*, suggesting there may be a similar regulatory relationship in *S. kowalevskii*. Expression begins around the prospective blastopore at the late blastula stage (Fig. S3Ci) and continues around the blastopore throughout later stages (Fig. S3C). An additional domain of expression is detected in a narrow band after gastrulation at a location that roughly corresponds to the domain of *wnt1* expression at later developmental stages (Fig. 2Civ) By later developmental stages, the expression at the anterior trunk is down regulated, and persists only in the posterior ectoderm.

**Sclerostin/WISE:** A subset of the cysteine knot family of genes is involved in regulating signaling ligands early in vertebrate development during body plan formation. These genes include; *gremlin*, *cerberus,* *DAN*, *WISE* and *sclerostin*. All have been implicated in inhibition of Bmp signaling [14]. WISE, and sclerostin also regulate Wnt signaling, and cerberus acts as an inhibitor of Bmp, Wnt and Nodal signaling [15]. There are few reports of orthologues of these proteins in protostomes [16], however, since sclerostin/WISE sequences are present in cnidarians, this absence from protostomes must be due to secondary loss. The family has expanded during vertebrate diversification. Functional studies of these proteins have been characterized during vertebrate development for their role in early axial patterning [14]. Two vertebrate cysteine knot proteins; SOST/sclerostin and WISE are closely related and seem to represent a vertebrate-specific duplication. *Sclerostin* is unusual in having a highly restricted expression pattern late in vertebrate development during the formation of bone, and no apparent earlier role. It has demonstrated activity in Bmp and Wnt repression [17,18,19]. WISE is closely related to sclerostin*,* and has context dependent anti Wnt and anti Bmp activity [20,21,22,23], and unlike *sclerostin*, is expressed during early development. The hemichordate orthologue of *WISE*/*sclerostin* groups strongly with *Nematostella* and Amphioxus *sclerostin*, outside the main grouping of vertebrate *sclerostin*/*WISE* genes (Fig. S1C). It is broadly expressed at late blastula stage (Fig. S3Di) in the animal hemisphere and with weaker expression at gastrula with detectable domains only in the posterior ectoderm (Fig. S3Dii). Then by 36 hrs of development, expression is broadly localized to the anterior two thirds of the embryo (Fig. S3Diii). Dorsal ectodermal midline expression is also detected, which is a similar localization to *bmp2/4* [24] (Fig. S3Div). Anterior endodermal expression is detected at the two groove stage. The expression domains in the anterior ectoderm and along the dorsal midline of the ectoderm are consistent with this protein potentially interacting with both Wnts and Bmps during axial patterning.

**R-spondin**: R-spondin is another secreted protein involved in the modulation of the Wnt/β-catenin pathway. Unlike the other genes discussed above, this protein has been implicated in the potentiation rather than antagonism of the signaling pathway. R-spondin interacts with the Wnt pathway by inhibiting Dkk-mediated internalization of LRP6, the Fz coreceptor [25,26,27]. Expression of *S. kowalevskii* *r-spondin* is first detected during gastrulation around the blastopore (Fig. S3Eii, iii), and then at later stages in the ectoderm at the boundary between collar and trunk, over the area where the first gill slit is beginning to form, and also at the boundary between the collar and proboscis (Fig. S3Eiv, v). Interestingly, these are areas where there is the most overlap of wnt ligand expression at similar developmental stages (Fig. 2, 3). During vertebrate development, *r-spondins* are also expressed in regions of active Wnt signaling. In the model presented by Binnerts et al. [27], R-spondin acts as a potentiator of β-catenin dependent gene expression, and its expression overlaps extensively with that of Wnt ligands. These data are consistent with a role of R-spondin in Wnt potentiation during the early development of *S. kowalevskii*.

Bibliography

1. Fedders H, Augustin R, Bosch TC (2004) A Dickkopf- 3-related gene is expressed in differentiating nematocytes in the basal metazoan Hydra. Dev Genes Evol 214: 72-80.

2. Guder C, Pinho S, Nacak TG, Schmidt HA, Hobmayer B, et al. (2006) An ancient Wnt-Dickkopf antagonism in Hydra. Development 133: 901-911.

3. Kawano Y, Kypta R (2003) Secreted antagonists of the Wnt signalling pathway. J Cell Sci 116: 2627-2634.

4. Niehrs C (2006) Function and biological roles of the Dickkopf family of Wnt modulators. Oncogene 25: 7469-7481.

5. Nakamura RE, Hackam AS Analysis of Dickkopf3 interactions with Wnt signaling receptors. Growth Factors.

6. Nakamura RE, Hunter DD, Yi H, Brunken WJ, Hackam AS (2007) Identification of two novel activities of the Wnt signaling regulator Dickkopf 3 and characterization of its expression in the mouse retina. BMC Cell Biol 8: 52.

7. Pinho S, Niehrs C (2007) Dkk3 is required for TGF-beta signaling during Xenopus mesoderm induction. Differentiation 75: 957-967.

8. Hsieh JC, Kodjabachian L, Rebbert ML, Rattner A, Smallwood PM, et al. (1999) A new secreted protein that binds to Wnt proteins and inhibits their activities. Nature 398: 431-436.

9. Gerlitz O, Basler K (2002) Wingful, an extracellular feedback inhibitor of Wingless. Genes Dev 16: 1055-1059.

10. Filmus J, Capurro M, Rast J (2008) Glypicans. Genome Biol 9: 224.

11. Piddini E, Vincent JP (2009) Interpretation of the wingless gradient requires signaling-induced self-inhibition. Cell 136: 296-307.

12. Traister A, Shi W, Filmus J (2007) Mammalian Notum induces the release of glypicans and other GPI-anchored proteins from the cell surface. Biochem J.

13. Torisu Y, Watanabe A, Nonaka A, Midorikawa Y, Makuuchi M, et al. (2008) Human homolog of NOTUM, overexpressed in hepatocellular carcinoma, is regulated transcriptionally by beta-catenin/TCF. Cancer Sci 99: 1139-1146.

14. Avsian-Kretchmer O, Hsueh AJ (2004) Comparative genomic analysis of the eight-membered ring cystine knot-containing bone morphogenetic protein antagonists. Mol Endocrinol 18: 1-12.

15. Piccolo S, Agius E, Leyns L, Bhattacharyya S, Grunz H, et al. (1999) The head inducer Cerberus is a multifunctional antagonist of Nodal, BMP and Wnt signals. Nature 397: 707-710.

16. Vitt UA, Hsu SY, Hsueh AJ (2001) Evolution and classification of cystine knot-containing hormones and related extracellular signaling molecules. Mol Endocrinol 15: 681-694.

17. Winkler DG, Sutherland MK, Geoghegan JC, Yu C, Hayes T, et al. (2003) Osteocyte control of bone formation via sclerostin, a novel BMP antagonist. EMBO J 22: 6267-6276.

18. Li X, Zhang Y, Kang H, Liu W, Liu P, et al. (2005) Sclerostin binds to LRP5/6 and antagonizes canonical Wnt signaling. J Biol Chem 280: 19883-19887.

19. van Bezooijen RL, Svensson JP, Eefting D, Visser A, van der Horst G, et al. (2007) Wnt but not BMP signaling is involved in the inhibitory action of sclerostin on BMP-stimulated bone formation. J Bone Miner Res 22: 19-28.

20. Itasaki N, Jones CM, Mercurio S, Rowe A, Domingos PM, et al. (2003) Wise, a context-dependent activator and inhibitor of Wnt signalling. Development 130: 4295-4305.

21. Guidato S, Itasaki N (2007) Wise retained in the endoplasmic reticulum inhibits Wnt signaling by reducing cell surface LRP6. Dev Biol 310: 250-263.

22. Shigetani Y, Itasaki N (2007) Expression of Wise in chick embryos. Dev Dyn 236: 2277-2284.

23. Lintern KB, Guidato S, Rowe A, Saldanha JW, Itasaki N (2009) Characterization of wise protein and its molecular mechanism to interact with both Wnt and BMP signals. J Biol Chem 284: 23159-23168.

24. Lowe CJ, Terasaki M, Wu M, Freeman RM, Jr., Runft L, et al. (2006) Dorsoventral patterning in hemichordates: insights into early chordate evolution. PLoS Biol 4: e291.

25. Kazanskaya O, Glinka A, del Barco Barrantes I, Stannek P, Niehrs C, et al. (2004) R-Spondin2 is a secreted activator of Wnt/beta-catenin signaling and is required for Xenopus myogenesis. Dev Cell 7: 525-534.

26. Kim KA, Wagle M, Tran K, Zhan X, Dixon MA, et al. (2008) R-Spondin family members regulate the Wnt pathway by a common mechanism. Mol Biol Cell 19: 2588-2596.

27. Binnerts ME, Kim KA, Bright JM, Patel SM, Tran K, et al. (2007) R-Spondin1 regulates Wnt signaling by inhibiting internalization of LRP6. Proc Natl Acad Sci U S A 104: 14700-14705.
