# Supplementary figures and images for: Anteroposterior axis patterning by early canonical Wnt signaling during hemichordate development
Source: PLoS Biol. 2018 Jan 16;16(1):e2003698. doi: 10.1371/journal.pbio.2003698 (PMC5786327; doi:10.1371/journal.pbio.2003698)

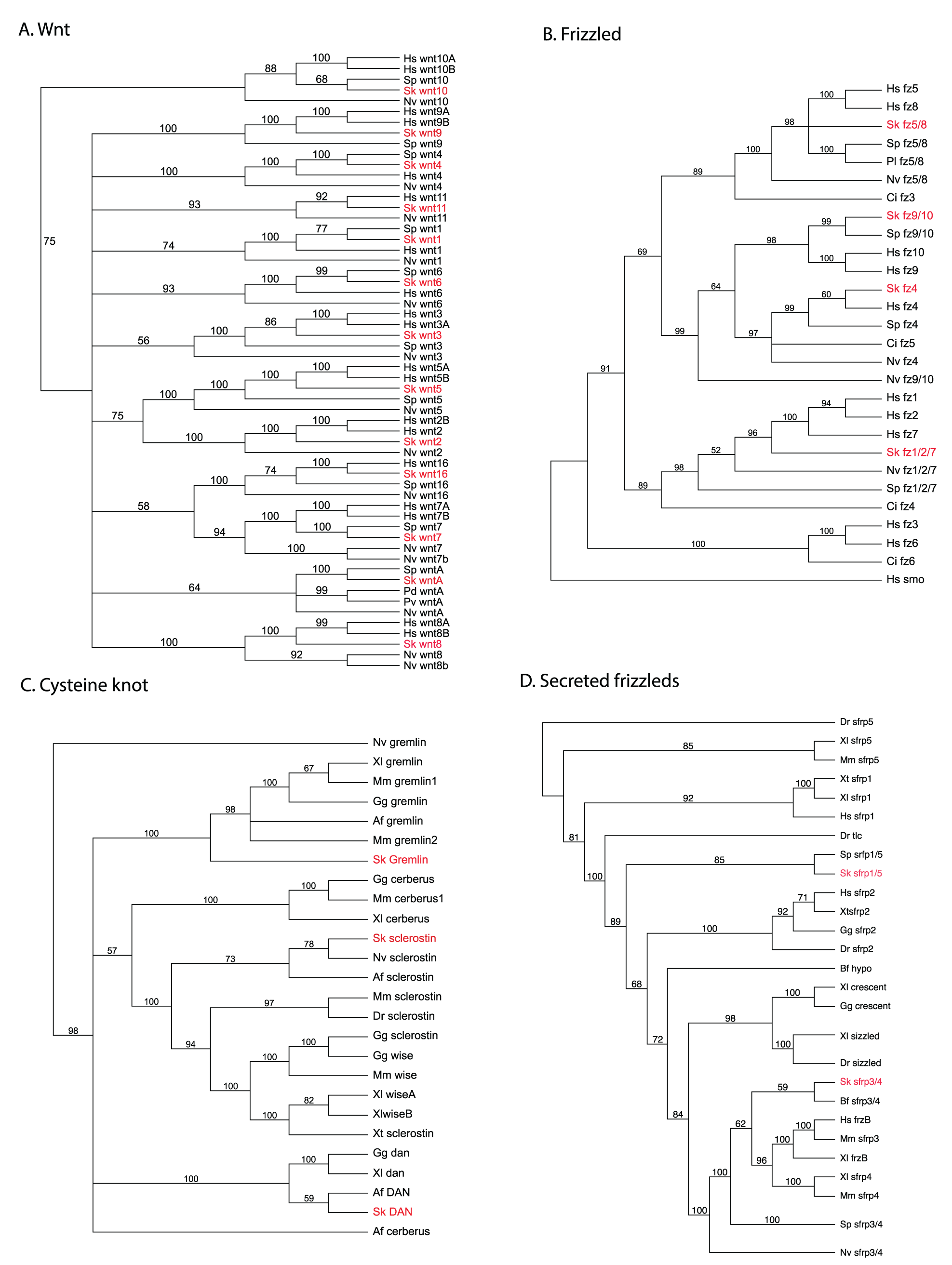

Supplement: S1 Fig — Bf, B. floridae; Ci, Ciona intestinalis; Dr, Danio rerio; Fz, frizzled; Gg, Gallus gallus; Hs, H. sapiens; Mm, M. musculus; Nv, Nematostella vectensis; Sfrp, secreted frizzled-related protein; Sk, S. kowalevskii; Sp, Strongylocentrotus purpuratus; Xl, X. laevis; Xt, X. tropicalis. (TIF) [file pbio.2003698.s006.tif]

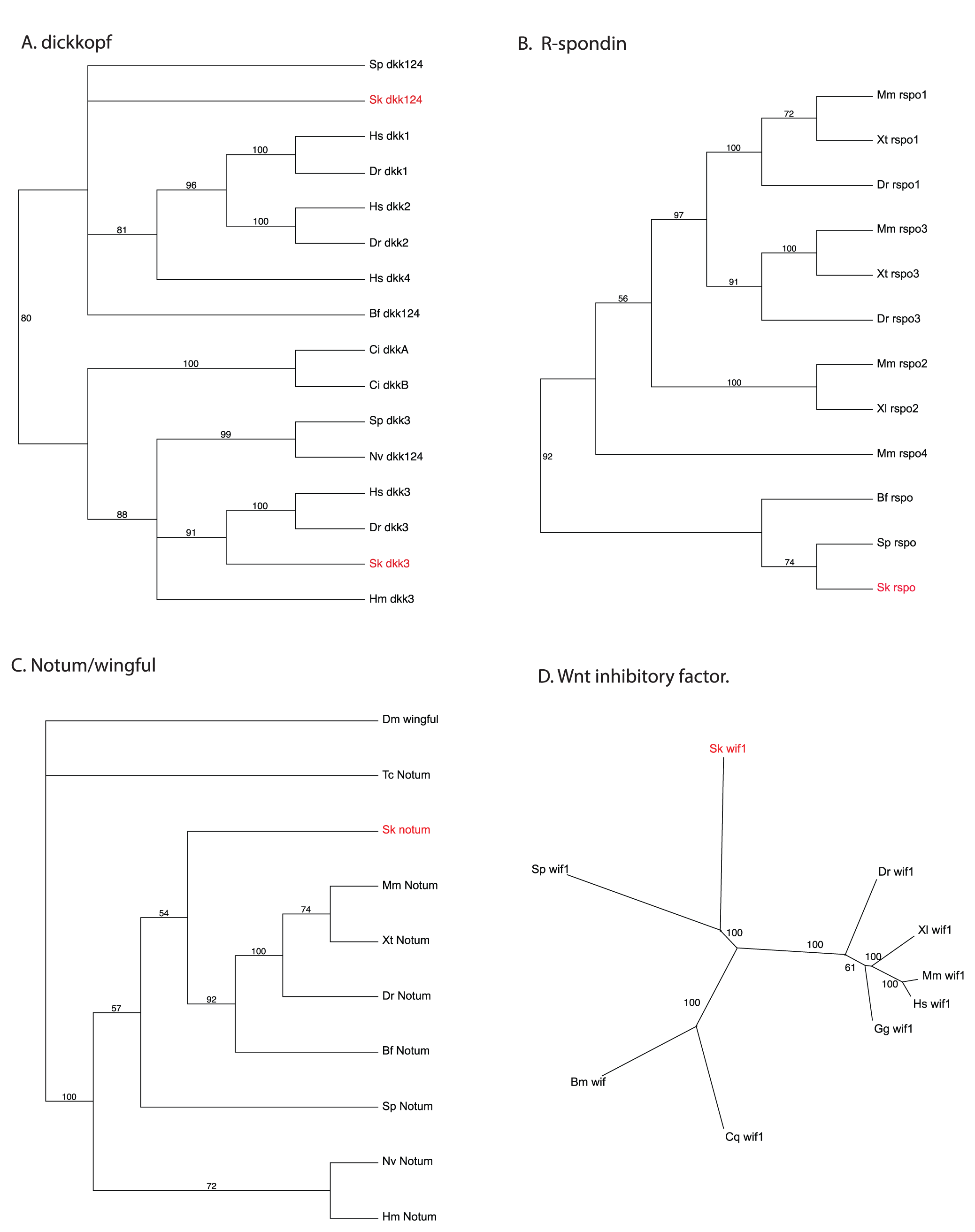

Supplement: S2 Fig — Bf, B. floridae; Bm, Bombyx mori; Ci, C. intestinalis; Cq, Culex quiquefasciatus; Dkk, Dickkopf; Dm, Drosophila melanogaster; Dr, Danio rerio; Gg, G. gallus; Hs, H. sapiens; Mm, Mus musculus; Nv, N. vectensis; Sk, S. kowalevskii; Sp, S. purpuratus; Tc, Tribolium castaneum; Wif, wnt inhibitory factor; Xl; X. laevis; Xt, X. tropicalis. (TIF) [file pbio.2003698.s007.tif]

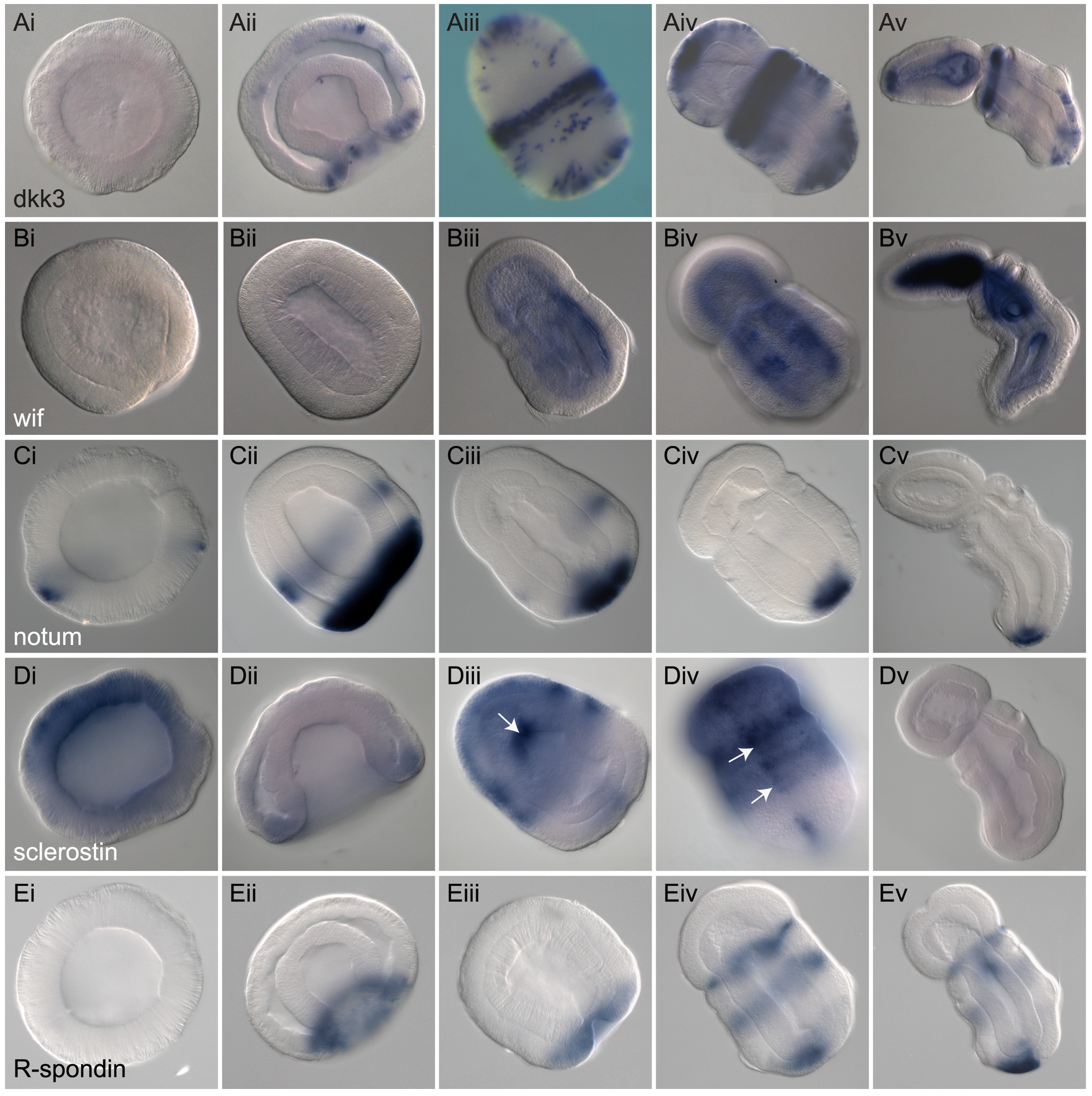

Supplement: S3 Fig — Whole mount in situ hybridization of Wnt modifier genes. All data are presented as optical sagittal or frontal sections following clearing in Murray Clear. Developmental staging is from blastula to 72 h of development. All embryos are oriented with anterior, or animal (in the case of blastula), to the top left of the panel and posterior, or vegetal, to the bottom right of the panel. Right column, ventral is to the bottom left. Unless otherwise noted, expression is ectodermal. (A), Expression of dkk3 at blastula (Ai), at late gastrula (Aii), at 48 h, surface view of an uncleared embryo (Aiii), at 60 h, side view (Aiv), and at 72 h of development in side view (Av). (B), Expression of wif at late gastrula (Bi), at 36 h (Bii), at 48 h (Biii-iv), and at 72 h of development (Bv). (C), Expression of notum at blastula (Ci), at late gastrula (Cii), at 48 h (Ciii), at 60 h, ventral view (Civ), and at 72 h of development (Cv). (D), Expression of sclerostin at blastula (Di), at early gastrula (Dii), at 36 h, white arrow indicates anterior endodermal expression (Diii), and at 60 h of development in dorsal view, with the focal plane through the dorsal ectoderm. Arrows indicate expression along the dorsal midline (Div), and at 72 h of development in side view (Dv). (E), Expression of r-spondin at blastula (Ei), at midgastrula (Eii), at late gastrula (Eiii), at 48 h in side view (Eiv), and at 60 h of development (Ev). wif, wnt inhibitory factor. (TIF) [file pbio.2003698.s008.tif]

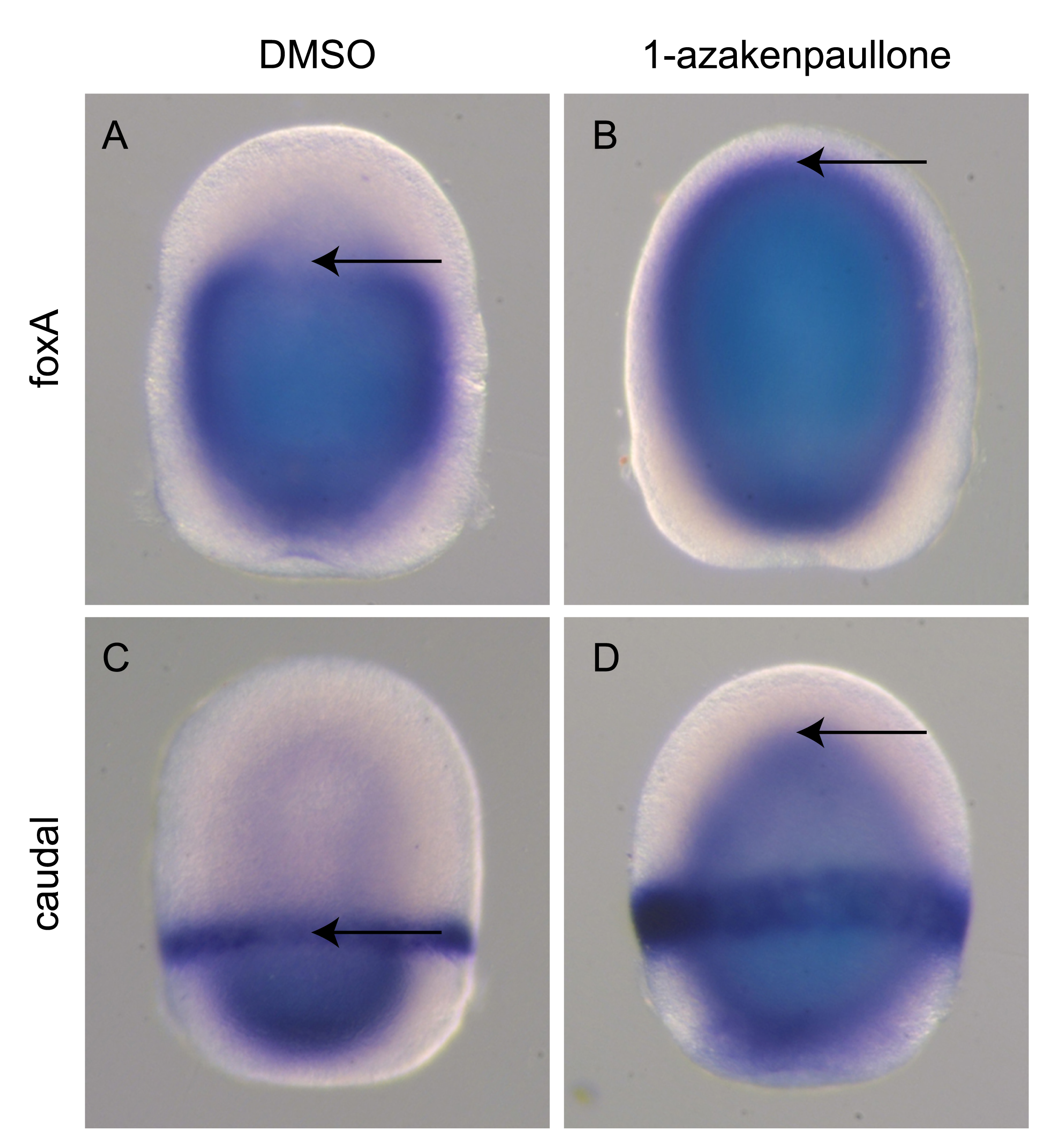

Supplement: S4 Fig — Embryos were treated with 10 μM of 1-azakenpaullone from midblastula stages (15.5 h) until fixation at 30 h of development. In situ hybridization for foxA (A and B) and caudal (C and D). Both markers show an anterior extension in their endomesodermal expression. DMSO-treated control embryos (A and C). 1-azakenpaullone-treated embryos (B and D). Anterior to the top. Arrows indicate the anterior limit of expression in the endomesoderm. cWnt, canonical Wnt; DMSO, dimethyl sulfoxide. (TIF) [file pbio.2003698.s009.tif]

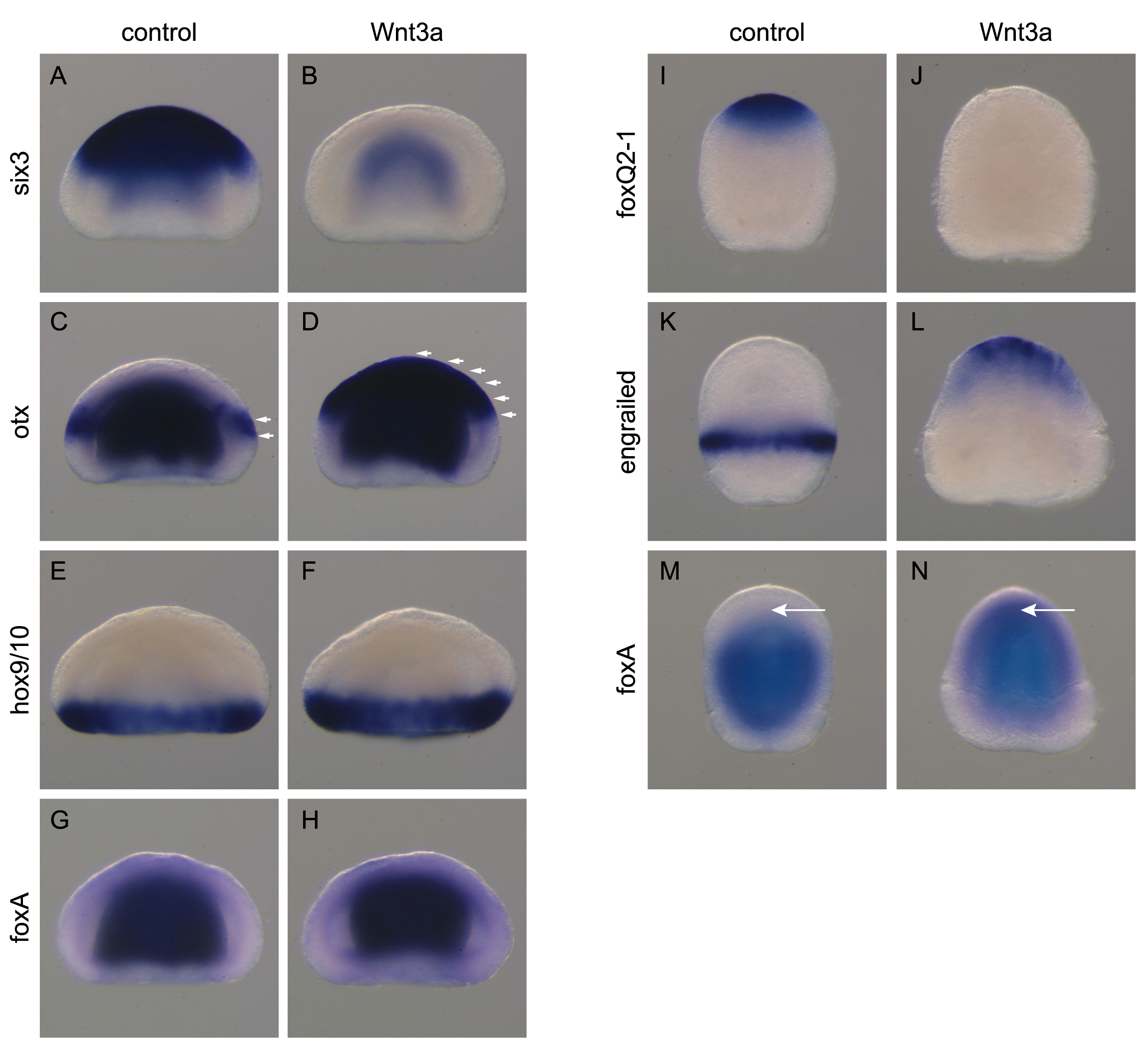

Supplement: S5 Fig — Embryos were treated with 200 ng/ml of Wnt3a protein from 1-cell stage to midgastrula stages, and fixed immediately (A-H) or at the end of gastrulation (I-N). Such a treatment led to a similar phenotype to what was observed upon wnt3 mRNA injection or 1-azakenpaullone treatment. Early endomesoderm specification was not affected as revealed by the internal expression of otx (C, D) and foxA (G, H), whereas the endodermal expression of foxA expanded into presumptive proboscis mesoderm at later stages (white arrow in M and N). Posterior ectodermal expression of hox9/10 was unchanged (E, F). Anterior ectodermal markers six3 (A, B) and foxQ2-1 (I, J) were repressed. Intermediate ectodermal markers expression was expanded and shifted anteriorly: ectodermal ring of otx (white arrows in C and D) and engrailed (K, L). Anterior is to the top. (TIF) [file pbio.2003698.s010.tif]

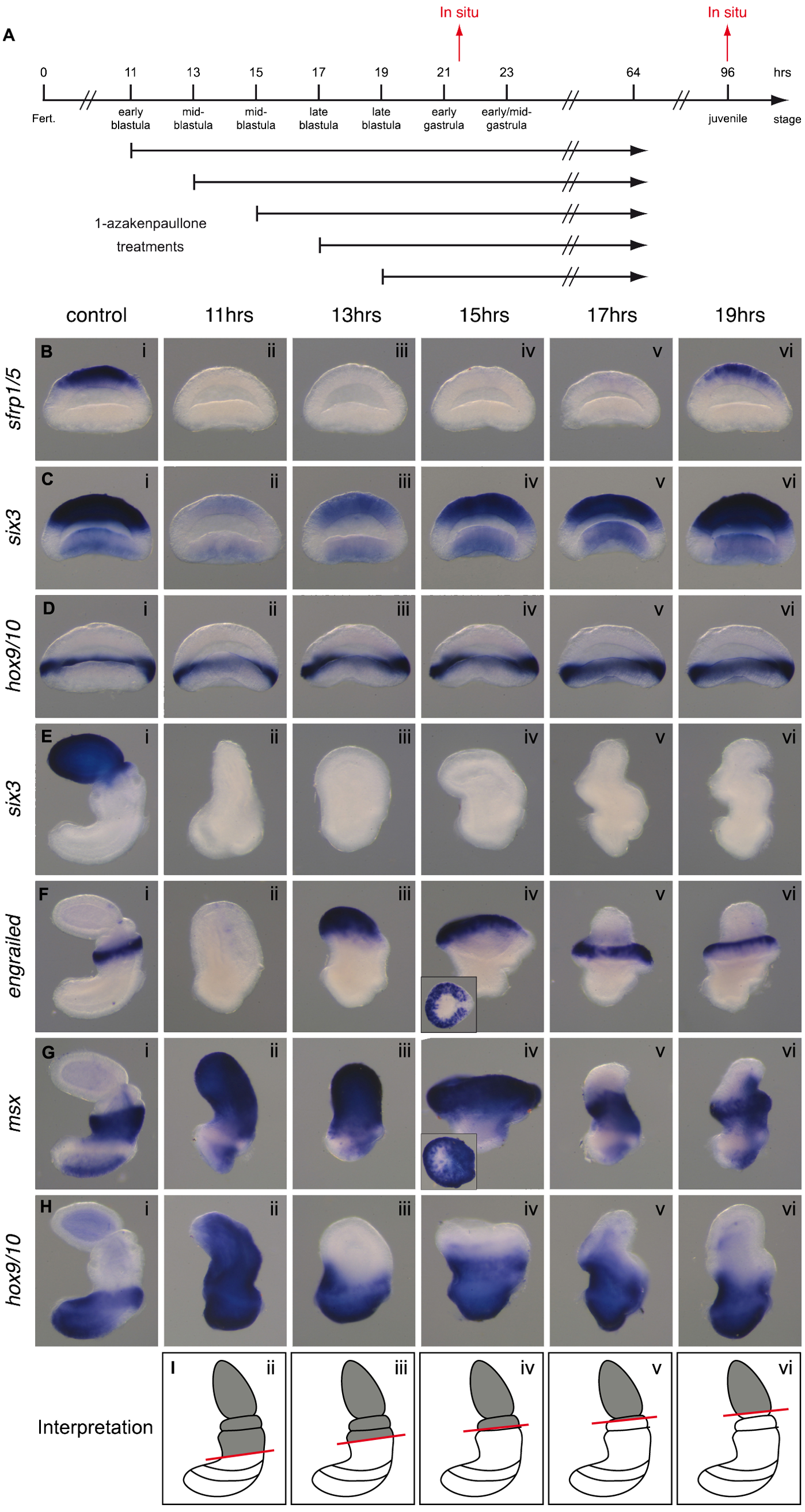

Supplement: S6 Fig — (A), Experimental scheme: embryos were treated with 10 μM of 1-azakenpaullone starting the treatment every 2 h and ending at 64 h of development. Embryos were fixed at early gastrula stages and at 96 h. (B-D), At gastrula stages, no morphological defects are detected, but ectodermal A/P marker expression is modified. While the expression of the anterior-most marker sfrp1/5 (B) is abolished, except for the latest treatment, in which a weaker expression is detected, the initial loss of the anterior marker six3/6 (C) is progressively recovered when the treatment is delayed. The posterior marker hox9/10 (D) expression is unchanged. (E-I), At four days of development, the morphology is dramatically affected; when embryos are treated early (11 h), they are truncated down to the anterior trunk. A full range of progressively less severe truncations are observed when the treatment is delayed until 19 h. Expression of the proboscis marker six3 (E), the anterior trunk marker en (F), the trunk marker msx (G), and the posterior trunk marker hox9/10 (H). (I), Schematic interpretation of the phenotypes; gray area corresponds to the truncated region. (i), DMSO-treated control embryos. 1-azakenpaullone treatment started at 11h (ii), 13 h (iii), 15 h (iv), 17h (v), and 19 h (vi). Gastrulae (B-D) are lateral view of hemi-sectioned embryos with animal to the top. Four days old embryos (E-H) are shown in lateral view with anterior to the top and ventral to the left, except inset in Fiv and Giv that show top views of the anterior of the embryo. A/P, anteroposterior; cWnt, canonical Wnt; DMSO, dimethyl sulfoxide. (TIF) [file pbio.2003698.s011.tif]

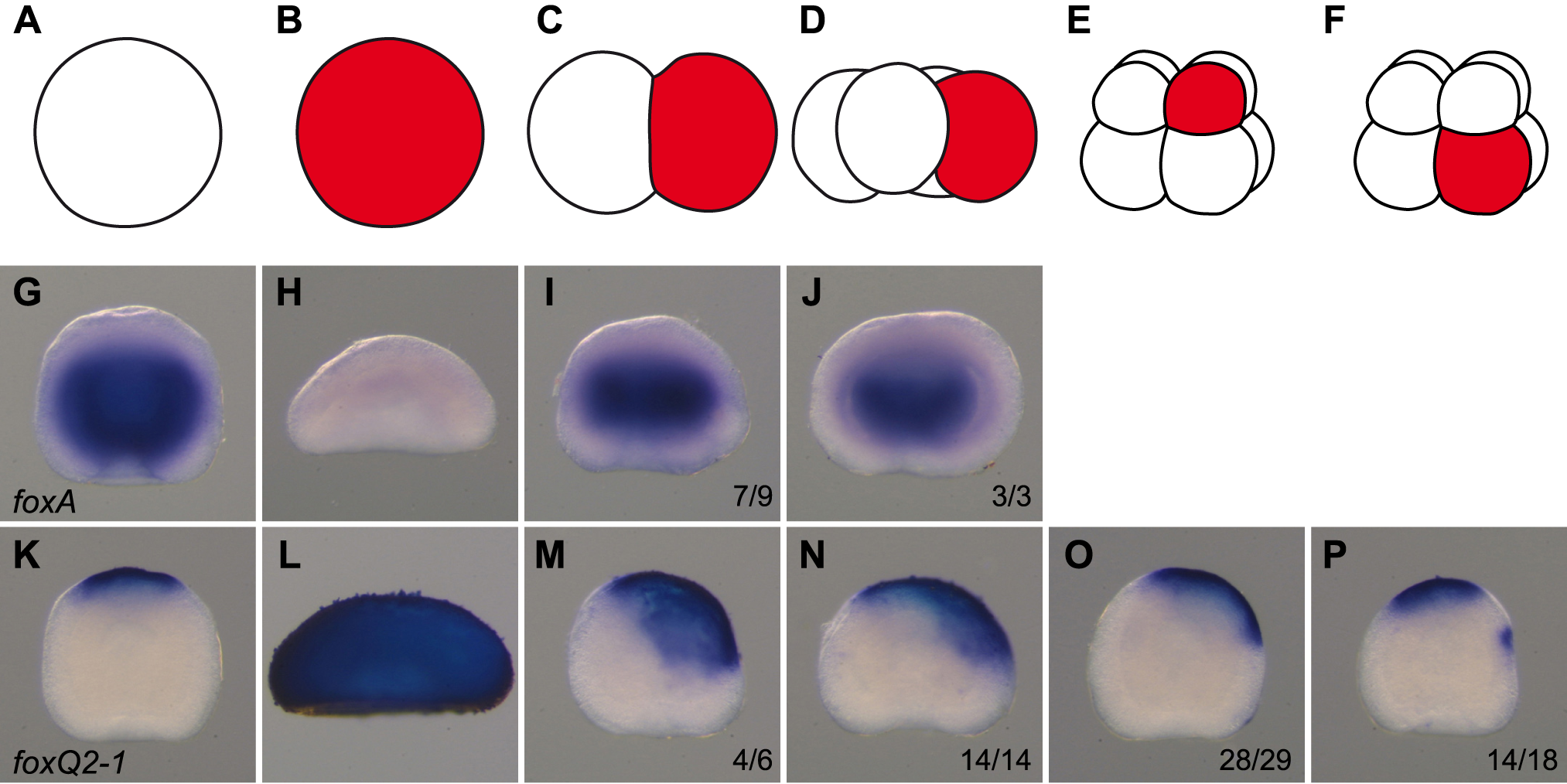

Supplement: S7 Fig — (A-F), Models of targeted injections, red indicating the targeted blastomere for injection. Data panels below each model represent embryos injected at 1-, 2-, 4-, or 8-cell stage. (G-J), Effect of injections on the endodermal marker foxA. (K-P), Effect of injection on the expression of the apical marker foxQ2-1. siRNA, short interfering RNA. (TIF) [file pbio.2003698.s012.tif]
